# Phosphorylation of CDC25C by AMP-activated protein kinase mediates a metabolic checkpoint during cell-cycle G_2_/M-phase transition

**DOI:** 10.1074/jbc.RA117.001379

**Published:** 2018-02-21

**Authors:** Yuqing Shen, John William Sherman, Xuyong Chen, Ruoning Wang

**Affiliations:** From the ‡ Center for Childhood Cancer and Blood Diseases, Hematology/Oncology and BMT, Research Institute at Nationwide Children's Hospital, Ohio State University, Columbus, Ohio 43205 and; the § Department of Microbiology and Immunology, Key Laboratory of Developmental Genes and Human Disease, Ministry of Education, Medical School, Southeast University, Nanjing 210009, China

**Keywords:** AMP-activated kinase (AMPK), mitosis, protein phosphorylation, cell cycle, metabolic regulation, CDC25C, metabolic checkpoint

## Abstract

From unicellular to multicellular organisms, cell-cycle progression is tightly coupled to biosynthetic and bioenergetic demands. Accumulating evidence has demonstrated the G_1_/S-phase transition as a key checkpoint where cells respond to their metabolic status and commit to replicating the genome. However, the mechanism underlying the coordination of metabolism and the G_2_/M-phase transition in mammalian cells remains unclear. Here, we show that the activation of AMP-activated protein kinase (AMPK), a highly conserved cellular energy sensor, significantly delays mitosis entry. The cell-cycle G_2_/M-phase transition is controlled by mitotic cyclin-dependent kinase complex (CDC2-cyclin B), which is inactivated by WEE1 family protein kinases and activated by the opposing phosphatase CDC25C. AMPK directly phosphorylates CDC25C on serine 216, a well-conserved inhibitory phosphorylation event, which has been shown to mediate DNA damage–induced G_2_-phase arrest. The acute induction of CDC25C or suppression of WEE1 partially restores mitosis entry in the context of AMPK activation. These findings suggest that AMPK-dependent phosphorylation of CDC25C orchestrates a metabolic checkpoint for the cell-cycle G_2_/M-phase transition.

## Introduction

Somatic cell-cycle progression involves a doubling and then equal distribution of cellular components and macromolecules into the two daughter cells. As such, interphase (G_1_, S, and G_2_ phases) represents a long period of cellular growth (accumulation of mass due to anabolic processes), whereas mitosis is the period of division, which is short and accompanied by metabolic suppression (1). Consequently, a fundamental problem in mammalian cells is coordination of the metabolic status with cell-cycle progression (2–6). The progression through the G_1_ phase in the mammalian cell cycle is regulated by growth factor/mitogen–mediated signals and metabolic status. The latter remotely resembles a mechanism in yeast known as START and represents a nutrient-sensing metabolic checkpoint (7–11). The signaling network behind the G_1_-phase metabolic checkpoint coordinates the cell-cycle machinery and metabolic activities, thus ensuring the availability of energy and nucleotide precursors for genome replication and a timely transition from G_1_ to S phase (12–15). Also, it has been suggested that a sufficient storage of energy and biosynthetic materials may enable the execution of mitosis in a robust and all-or-none fashion (16–19). It is conceivable that a cell size–sensing mechanism may play a role in coordinating metabolic status (growth) and the G_2_/M-phase transition. This mechanism would allow cells to keep biosynthetic activity in check, ensuring sufficient biomass accumulation to produce daughter cells with the proper size (20–24). These studies implicate the existence of metabolic checkpoints during the G_1_/S- and G_2_/M-phase transition.

The AMP-activated protein kinase (AMPK)^2^ complex is a central signaling node that keeps the cellular metabolic status in check by sensing changes in cellular AMP and other cellular metabolites, indicative of energy and nutrient status. Upon its activation, AMPK acts to maintain ATP homeostasis by rewiring metabolic programs to produce more energy and meanwhile suppressing many energy-consuming cellular processes, including cell-cycle progression (25, 26). It has been known that AMPK activation inhibits cell proliferation by increasing p21 and p27, two inhibitors of cyclin-dependent kinase (CDK) complex. Under conditions of insufficient nutrients, such as low glucose in cell culture medium, AMPK phosphorylates transcription factor p53, and this phosphorylation event mediates the suppression of G_1_-phase progression under glucose restriction (27–30). The mammalian target of rapamycin (mTOR), an evolutionarily conserved protein kinase, integrates environmental cues to coordinately regulate many fundamental cellular processes, including cell-cycle progression through the G_1_ phase. AMPK has been reported to directly phosphorylate key components of mTORC1 and consequently suppress mTORC1 signaling and the G_1_/S-phase transition (31–36). These findings clearly implicate AMPK as a key player in coupling the cellular metabolic status to the regulation of the G_1_/S-phase transition. However, the robustness of AMPK-dependent regulation on a myriad of fundamental cellular processes in response to metabolic stress suggests the presence of additional regulatory steps coupling AMPK and cell-cycle progression, and the molecular mechanisms behind these unrevealed regulatory steps remain to be explored.

The G_2_/M-phase transition is driven by a series of tightly regulated and coordinated signaling events that eventually lead to the activation of CDC2-cyclin B (37–40). Among these events, the rate-limiting step in directing mitosis entry is the activation of dual-specificity protein phosphatase CDC25C. The activation of CDC25C generally involves two steps, initiation and amplification (41, 42). The latter requires an array of protein kinases that can extensively phosphorylate CDC25C and change its conformation (43–50). Likewise, the initiation step of CDC25C activation requires multiple coordinated events, including dephosphorylation of serine 216, a conserved inhibitory phosphorylation, dissociation from the inhibitor 14-3-3, and change in the subcellular location (51–55). The amplification step of CDC25C activation is part of a positive-feedback loop that enables a rapid, robust, and irreversible mitosis entry, whereas the initiation step represents a surveillance mechanism that ensures the order and integrity of the cell-cycle machinery (56). Supporting this idea, the DNA damage–induced G_2_-phase checkpoint is largely mediated through inhibition of CDC25C, thus suppressing CDC2-cyclin B. Importantly, this is a p53-independent mechanism that is critical for the DNA damage response in most cancer cells because p53 loss of function is common in cancer cells (57–60). Because metabolic stress also causes cell-cycle arrest, it is conceivable that CDC25C may also represent a critical target of the metabolic checkpoint on cell-cycle progression.

In this study, we report a crucial role of AMPK in regulating the G_2_/M-phase transition. Unlike AMPK-dependent regulation on the G_1_/S transition, AMPK activation delays mitosis entry independently from its regulation on p21, p27, and mTORC1. Instead, AMPK directly phosphorylates CDC25C on serine 216, an inhibitory phosphorylation event that has been previously shown to retain CDC25C in the cytosol and keep it inactive (51, 53, 54, 61, 62). Either acute overexpression of CDC25C-S216A mutant or inhibition of WEE1 can reverse cell-cycle G_2_-phase arrest imposed by AMPK activation. Moreover, pharmacologic abrogation of AMPK-mediated cell-cycle arrest by WEE1 inhibitor induces cell death. These findings reveal a novel AMPK-dependent metabolic checkpoint on cell-cycle G_2_/M transition, and pharmacological abrogation of this checkpoint may represent a new therapeutic approach to treat cancers.

## Results and discussion

### Activation of AMPK at G_2_ phase delays mitosis entry

**Figure 1.**
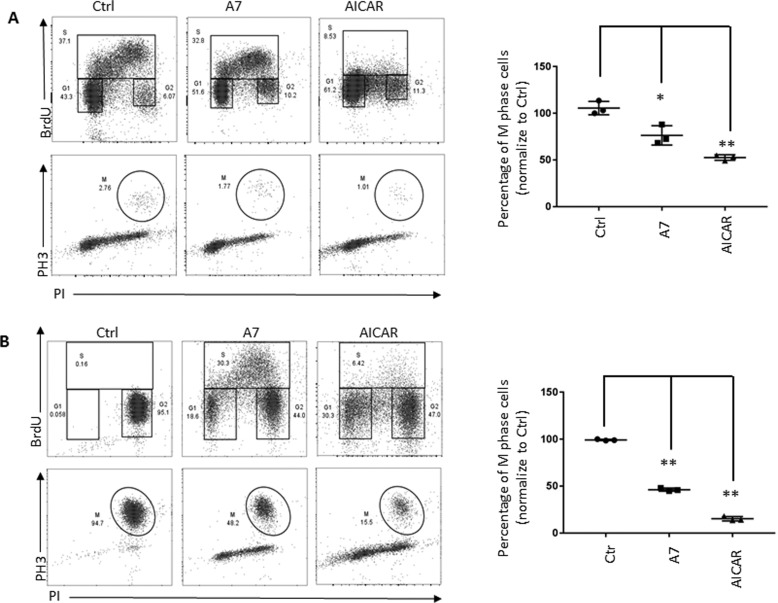
**Pharmacologic activation of AMPK increases the percentage of cells in G_1_ and G_2_ phase.** *A*, HeLa cells were treated with 300 μm A7 or 2 mm AICAR for 14 h before harvesting the samples. The percentage of cells in G_1_, S and G_2_ phase is indicated by PI staining in combination with BrdU incorporation (*top*). The percentage of cells in mitosis (*M*) is indicated by phosphorylation of histone H3 (*pH3*, *bottom*). The *scatter plot* indicates the percentage of M-phase cells treated with AMPK activator, and the percentage of M-phase cells in vehicle-treated cells was set to 100. *Error bars*, S.D. of triplicate samples. *B*, HeLa cells were treated with 300 μm A7 or 2 mm AICAR in combination with 100 ng/ml nocodazole for 14 h. The percentage of cells in G_1_, S, and G_2_ phase is indicated by PI staining and BrdU incorporation (*top*). The percentage of M-phase cells is indicated by phosphorylation of histone H3 (*pH3*, *bottom*). The *scatter plot* indicates the percentage of M-phase cells treated with AMPK activator, and the percentage of M-phase cells in vehicle-treated cells was set to 100. *Error bars*, S.D. of triplicate samples. *, *p* < 0.05; **, *p* < 0.01.

Previous studies have demonstrated an AMPK-dependent cell-cycle checkpoint at the G_1_/S-phase boundary, which may ensure the coordination of DNA synthesis in S phase with the availability of nutrients for nucleotide biosynthesis in G_1_ phase (27, 33). However, it is still unclear whether the G_2_/M-phase transition is regulated by AMPK and represents a checkpoint for the coordination of cell metabolism and cell-cycle progression. For this, we treated HeLa cells overnight with two mechanistically distinct pharmacologic activators of AMPK, 5-aminoimidazole-4-carboxamide 1-β-d-ribofuranoside (AICAR) or A 769662 (A7) (63). AICAR is considered as an AMP-mimetic compound that directly binds to a nucleotide-binding pocket in the AMPKα subunit and promotes AMPK kinase activities; A7 binds to a cleft between the AMPKα and β subunits and causes allosteric activation of the AMPK kinase complex (63–66). We found that both AICAR and A7 increased the percentage of cells in the G_1_ and G_2_ phases, as indicated by PI staining in combination with BrdU incorporation (Fig. 1*A*). By contrast, the percentage of cells in mitosis indicated by phosphorylation of histone H3 (*pH3*) is reduced following AICAR and A7 treatment (Fig. 1*A*). Notably, the disappearance of BrdU incorporation in AICAR group is probably due to the substrate competition between BrdU and AICAR, both of which are nucleotide analogs. Next, we repeated the experiment in the presence of nocodazole, a reversible inhibitor of microtubule polymerization, which blocks mitosis exit and therefore highlights the changes of mitotic entry following treatments. Both AICAR and A7 reduced the percentage of cells in mitosis compared with the control group (Fig. 1*B*).

**Figure 2.**
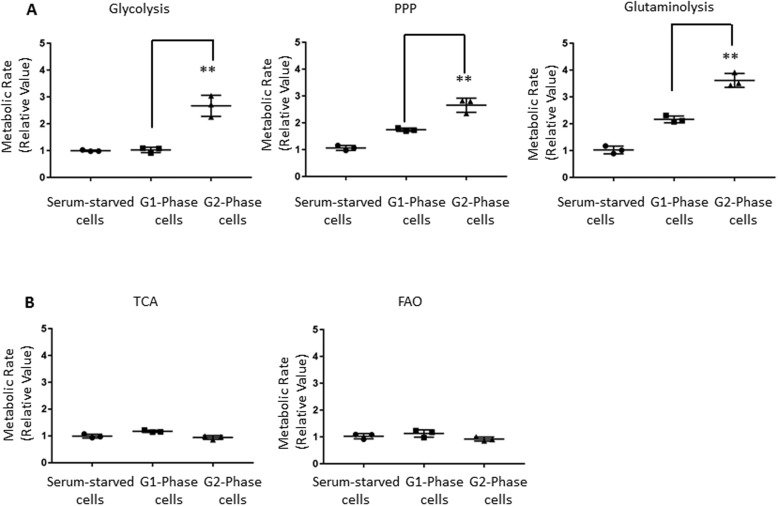
**Cells display heightened glucose and glutamine catabolism in G_2_ phase.** *A*, HeLa cells were released into G_1_ phase after mitotic shake-off or were release into G_2_ phase after double-thymidine arrest. Unsynchronized HeLa cells incubated in medium without serum for 6 h before the assay were used as control for the baseline metabolic activity. The indicated metabolic activities were determined by measuring the generation of ^3^H_2_O from [5-^3^H]glucose (glycolysis; *left*) or the generation of ^14^CO_2_ from [1-^14^C]glucose (PPP; *middle*) or from [U-^14^C]glutamine (glutaminolysis; *right*). Metabolic rate was normalized to serum-starved cells. *Error bars*, S.D. of triplicate samples. *B*, indicated metabolic activities were determined by measuring the generation of ^14^CO_2_ from [2-^14^C]pyruvate (TCA; *left*) and measuring the generation of ^3^H_2_O from [9,10-^3^H]palmitic acid (fatty acid β-oxidation; *right*). Metabolic rates were normalized to control. *Error bars*, S.D. of triplicate samples. **, *p* < 0.01.

We next applied radiochemical-based approaches to determine the activity of major catabolic pathways that could fuel the biosynthetic programs in cells released into G_1_ phase or G_2_ phase. We also included cells starved by serum removal as a control to indicate the baseline metabolic activity. Compared with cells at G_1_ phase or serum-starved cells, cells at G_2_ phase significantly up-regulated glycolysis, indicated by the detritiation of [5-^3^H]glucose; glucose consumption via the pentose phosphate pathway (PPP), indicated by ^14^CO_2_ release from [1-^14^C]glucose; and glutamine consumption through oxidative catabolism (glutaminolysis), indicated by ^14^CO_2_ release from [U-^14^C]glutamine (Fig. 2*A*). In contrast, both mitochondria-dependent pyruvate oxidation through the tricarboxylic acid (TCA) cycle, indicated by ^14^CO_2_ release from [2-^14^C]pyruvate, and fatty acid β-oxidation, indicated by the detritiation of [9,10-^3^H]palmitic acid, were comparable among all three groups (Fig. 2*B*). These data suggest that cells at G_2_ phase actively engage glucose and glutamine catabolic programs to meet their bioenergetic and biosynthetic demands.

**Figure 3.**
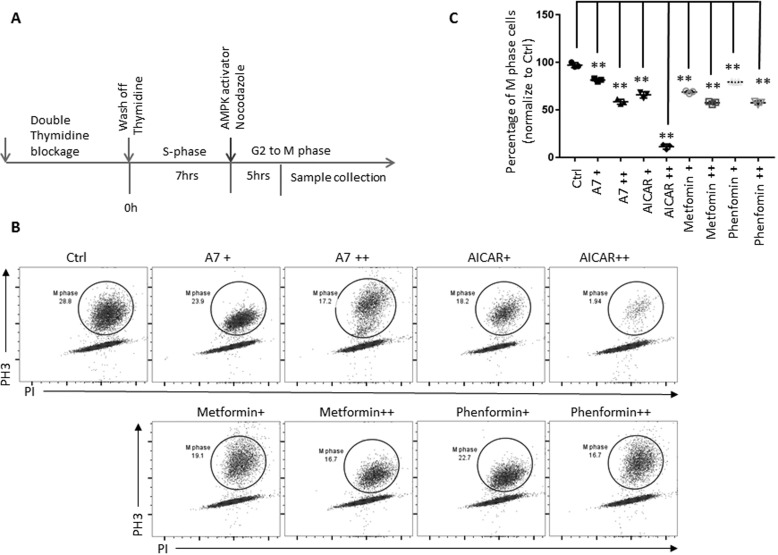
**Acute activation of AMPK at G_2_ phase delays mitosis entry.** *A*, *schematic view* of cell synchronization and the indicated treatments. Representative flow cytometric *dot plots* (*B*) and the scatter plot (*C*) indicate the percentage of M-phase cells treated with indicated compounds at G_2_ phase (A7+, 150 μm; A7++, 300 μm; AICAR+, 2 mm; AICAR++, 4 mm; metformin+, 2 mm; metformin++, 4 mm; phenformin+, 20 μm; phenformin++, 100 μm). The percentage of M-phase cells in vehicle-treated cells was set to 100. *Error bars*, S.D. of triplicate samples. **, *p* < 0.01.

Next, we sought to determine whether the acute activation of AMPK at G_2_ phase would cause a delay of mitosis entry. This would determine whether the delay of mitosis entry is a secondary effect from the G_1_/S-phase transition in the presence of AMPK activators. For this, we first synchronized cells at the G_1_/S boundary by double thymidine blockage and then released the cells into S phase and treated them with AMPK activators and nocodazole once they reached G_2_ phase (Fig. 3*A*). In addition to AICAR and A7, we also included metformin and phenformin, two respiration chain complex I inhibitors, to indirectly activate AMPK by suppressing ATP production (67). Our results clearly showed that these AMPK activators reduced the percentage of cells in mitosis in a dose-dependent manner (Fig. 3, *B* and *C*). Moreover, none of the acute treatment with these compounds caused significant apoptosis, as measured by PI uptake and cell-surface annexin V staining (Fig. S1). Collectively, our results suggest that activation of AMPK in cells at G_2_ phase delays mitosis entry.

### DNA damage pathway and mTOR pathway are not involved in mediating AMPK-dependent regulation on G_2_/M-phase transition

**Figure 4.**
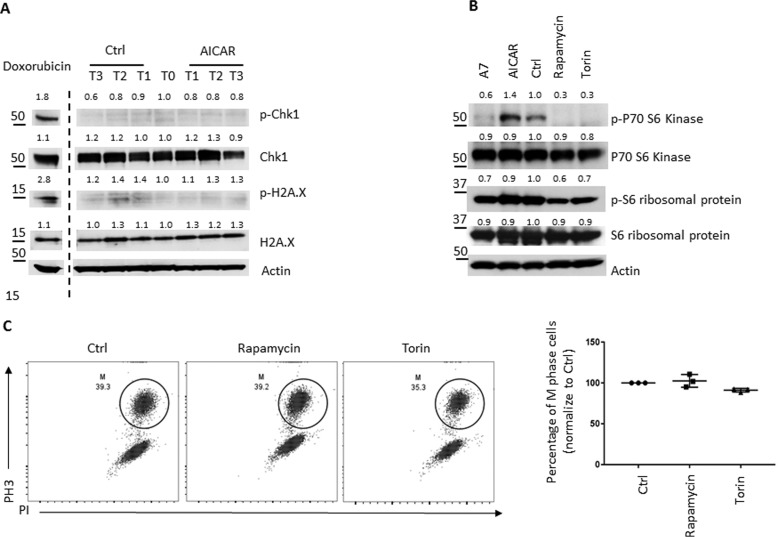
**Pharmacological activation of AMPK on cells in G_2_ phase does not activate the DNA damage pathway or suppress the mTOR pathway.** *A*, HeLa cells were treated with or without 2 mm AICAR at G_2_ phase alone with nocodazole. Cells were collected at different time points (T0, 7 h after the second thymidine release when AMPK activators and nocodazole were added; T1, 2 h after T0; T2, 4 h after T0; T3, 6 h after T0). The level of the indicated proteins in HeLa cells was determined by immunoblotting. Doxorubicin (1 μg/ml), as a DNA damage-inducing agent, was included as a positive control. *B*, HeLa cells were treated with 300 μm A7, 2 mm AICAR, 200 nm rapamycin, or 2 μm torin at G_2_ phase alone with nocodazole. Cells were collected 12 h after the second thymidine release. The level of the indicated proteins in HeLa cells was determined by immunoblot. *C*, the percentage of M-phase cells with the indicated treatments was determined by pH3 staining. The representative flow cytometric dot plots (*left*) and scatter plot (*right*) indicate the percentage of M-phase cells in different groups. The percentage of M-phase cells in vehicle-treated cells was set to 100. *Error bars*, S.D. of triplicate samples.

It has been well-established that cells in G_2_ phase with damaged DNA are prevented from entering into mitosis, and the control mechanisms behind this are known as the G_2_ checkpoint (60, 68–71). To determine whether activation of AMPK cross-talks with the DNA damage pathway and causes G_2_ arrest, we treated cells with AICAR at G_2_ phase and examined molecules involved in the DNA damage response pathways in cells collected at various time points. Doxorubicin, a reagent that causes DNA adducts and activates the DNA damage response, readily induced phosphorylation of checkpoint kinase 1 (Chk1) and histone H2AX (H2AX), two characteristic biomarkers of the DNA damage response (72). However, treatment with AICAR failed to induced any visible phosphorylation of Chk1 and H2AX (Fig. 4*A*). Previous studies have demonstrated that AMPK phosphorylates p53 and causes the accumulation of p21, a transcriptional target of p53 that mediates p53-dependent regulation on cell cycle (28, 73–75). In a different cellular context, AMPK was reported to regulate p27 and induce autophagy (29). Both p21 and p27 are CDK inhibitors and play critical roles in cell-cycle regulation (76, 77). However, we have found that the treatment with AICAR failed to induce the expression of p21 or p27 (Fig. S2). Our findings are also consistent with earlier reports showing no detectable p53 protein in HeLa cells (78, 79).

It has also been reported that AMPK regulates cell-cycle progression through directly inhibiting mTORC1 complex, which is the central player in sensing nutrients and coordinately regulating cell-cycle progression (31, 33–36). To determine whether mTORC1 is involved in mediating AMPK-dependent regulation on G_2_/M transition, we treated cells with either AMPK activators or mTORC1 inhibitors at G_2_ phase and examined the phosphorylation of mTORC1 effector molecules, P70 kinase and ribosomal protein S6, in cells collected at the time when control cells start entering into mitosis. Whereas A7 treatment and the mTORC1 inhibitor treatments led to the suppression of mTORC1, as indicated by the loss of phosphorylation of P70 kinase and S6, AICAR treatment failed to suppress mTORC1 activities (Fig. 4*B*). Importantly, neither rapamycin nor torin treatment delayed mitosis entry (Fig. 4*C*). Collectively, these findings suggested that mTORC1 is not involved in mediating AMPK-dependent regulation of the G_2_/M transition.

### AMPK phosphorylates CDC25C at serine 216 in vitro and in cells

**Figure 5.**
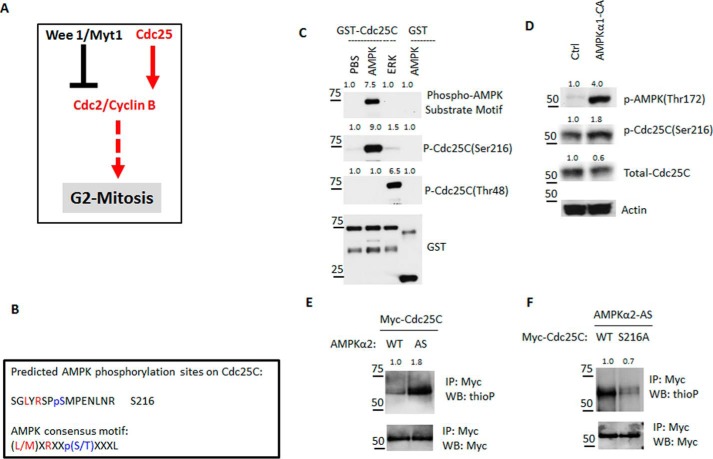
**AMPK phosphorylates CDC25C at serine 216 *in vitro* and in cells.** *A*, model showing that G_2_/M-phase transition is regulated by CDC25C and WEE1. *B*, potential AMPK phosphorylation motif of CDC25C was predicted by Scansite3. The AMPK consensus phosphorylation motif and the predicted motif are shown in *single-letter amino acid code*. Positions marked as *red* showed the similarity between the two motifs, and the predicted phosphorylation site of CDC25C is marked as *blue. C*, bacterially expressed GST-CDC25C or GST was incubated with recombinant AMPK or ERK in an *in vitro* phosphorylation assay. Proteins were resolved by SDS-PAGE and immunoblotting for the indicated antibodies. *D*, HeLa cells were transfected with a construct expressing a constitutively active mutant of the AMPK catalytic subunit (AMPK-CA) or a blank control plasmid for 2 days, and the expression of the indicated proteins in transfected cells was determined by immunoblot. *E*, analog-sensitive kinase assay in cells. HeLa cells were transfected with Myc-CDC25C together with either WT-AMPKα2 or AS-AMPKα2 and treated with ATPγS analog as a phosphodonor. CDC25C was immunoprecipitated (*IP*) with Myc tag antibody and blotted (*WB*) with the indicated antibodies. *F*, analog-sensitive kinase assay in cells. HeLa cells were transfected with AS-AMPKα2 together with either WT or S216A mutant Myc-CDC25C and treated with ATPγS analog as a phosphodonor. CDC25C was immunoprecipitated with Myc tag antibody and blotted with the indicated antibodies.

The activity of CDKs oscillates throughout the cell cycle and determines the transition between different cell-cycle phases (76, 77). Mitosis entry is driven by CDC2-cyclin B, which is coordinately regulated by CDC25, WEE1, and MYT1. WEE1 and MYT1 phosphorylate and keep CDC2-cyclin B inactive in G_2_ phase. CDC25 activates CDC2-cyclin B and drives cells into mitosis by dephosphorylating the inhibitory phosphorylation sites on CDC2-cyclin B (Fig. 5*A*). Given that the mitotic regulators mentioned above are all regulated by phosphorylation and AMPK is a signaling protein kinase, we asked whether AMPK is capable of directly phosphorylating and modulating any of the above mitotic regulators. Previous studies have revealed the optimal consensus phosphorylation motif for AMPK (33, 80). We therefore applied a bioinformatics tool (Scansite3 (81)) to search for putative AMPK-mediated phosphorylation motifs in CDC25C, WEE1, MYT1, CDC2, and cyclin B1. The inspection of these protein sequences revealed one putative AMPK phosphorylation site, serine 216, in CDC25C (Fig. 5*B*). The phosphorylation of serine 216 plays a key role in the temporal and spatial regulation of CDC25C and the G_2_/M transition in response to DNA damage and during normal cell-cycle progression (51, 53, 54, 61, 62). To validate serine 216 as an AMPK phosphorylation site in CDC25C, we took an integrated stepwise approach. We first transfected cells with constructs expressing either WT or mutant CDC25C (serine to alanine/S216A) fused to the Myc epitope tag (Myc-CDC25C), immunoprecipitated CDC25C with anti-Myc epitope tag antibodies, and then immunoblotted the protein samples using a phosphorylation-specific antibody that recognizes the phosphorylated AMPK consensus motif (33, 80). Whereas the phospho-AMPK substrate motif antibody recognized WT CDC25C, the S216A mutation abolished such recognition, suggesting that Ser-216 is within the AMPK substrate motif in CDC25C (Fig. S3*A*). Second, we generated recombinant CDC25C fused to glutathione *S*-transferase (GST-CDC25C) and a non-related GST-tagged protein and phosphorylated these proteins with recombinant AMPK or extracellular signal-regulated kinase 1 (ERK1) or PBS control. ERK readily phosphorylated CDC25C at threonine 48, as we reported previously (49). However, only the AMPK-mediated phosphorylation site in CDC25C was recognized by the phospho-AMPK substrate motif antibody or the pSer-216 antibody (Fig. 5*C*). Third, we treated cells with AICAR or transfected cells with either a control construct or a construct expressing a constitutively active mutant of the AMPK catalytic subunit (AMPK-CA) (82) and then immunoblotted with the pSer-216 antibody. Both pharmacologic and genetic activation of AMPK readily enhanced the phosphorylation of Ser-216 in CDC25C (Fig. 5*D* and Fig. S3*B*). Finally, we applied a chemical genetic approach to validate Ser-216 in CDC25C as a genuine AMPK-mediated phosphorylation site in cells (83–85). This approach is based on the concept that protein kinase can only use ATP as a phosphate donor; however, the point mutation of a conserved gatekeeper residue in the ATP-binding pocket of a protein kinase would allow mutant protein kinase (analog-specific (AS)) to utilize ATP analog (ATPγS) as a phosphate donor. The specific labeling of kinase substrate with thiophosphate (thioP), followed by protein immunoprecipitation and alkylation, would enable the recognition of AS mutant–dependent phosphorylation with a thioP-specific antibody. This approach has been applied to identify direct substrates of various protein kinases, including AMPK (83, 86). Thus, we co-transfected cells with a construct expressing Myc-tagged CDC25C as well as a construct expressing either FLAG-tagged WT or AS-AMPK and showed that Myc-tagged CDC25C could be labeled with thioP only in the presence of AS-AMPK (Fig. 5*E*). Then we followed up the above experiment by co-transfecting cells with a construct expressing AS-AMPK as well as a construct expressing either Myc-tagged WT or S216A CDC25C. The recognition by thioP-specific antibody was significantly compromised by S216A mutation (Fig. 5*F*), suggesting that Ser-216 is a direct phosphorylation site and very likely the only phosphorylation site of AMPK on CDC25C.

### Acute modulation of CDC25C or WEE1 partially relieves AMPK-dependent inhibition of the G_2_/M-phase transition

**Figure 6.**
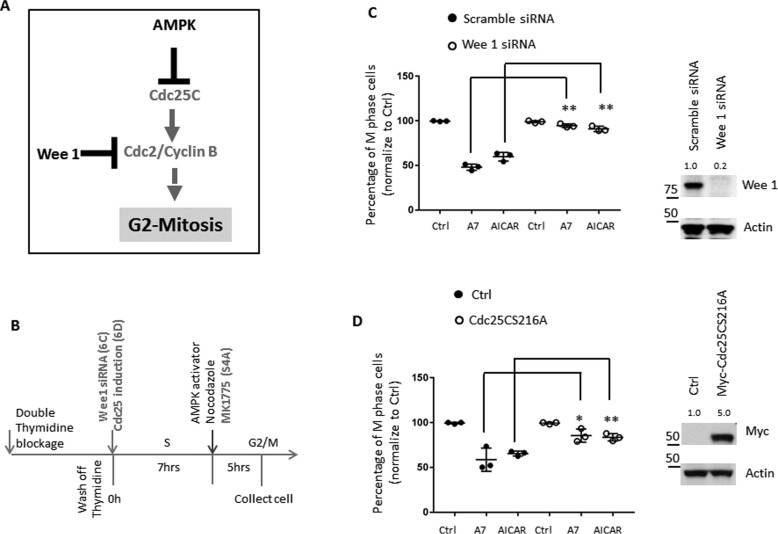
**Acute induction of CDC25C or suppression of WEE1 partially reverses the effect of AMPK activation on the G_2_/M-phase transition.** *A*, model showing that the G_2_/M-phase transition is regulated by CDC25C and WEE1. *B*, *schematic view* of cell synchronization and the indicated treatments. Synchronized HeLa cells that stably express reverse tetracycline-controlled transactivator and doxycycline-inducible CDC25C were treated with doxycycline when cells were released from the second thymidine block (G_1_/S boundary). Synchronized HeLa cells were transfected with WEE1 siRNA at the G_1_/S boundary or treated with WEE1 inhibitor at G_2_ phase (7 h after cells were released from the second thymidine block), respectively. AMPK activators and nocodazole were added when cells are in G_2_ phase. *C*, the percentage of M-phase cells in HeLa cells transfected with scramble siRNA or WEE1 siRNA was determined by phosphorylation of histone H3 (*pH3*) staining. The percentage of M-phase cells in vehicle-treated cells was set to 100, and the value of AMPK activator–treated cells was normalized to that of vehicle-treated cells. *Error bars*, S.D. of triplicate samples (*left*). Cell lysates were blotted with the indicated antibodies (*right*). *D*, the percentage of M-phase cells in HeLa cells that stably express reverse tetracycline transcriptional activator (*Ctrl*) or CDC25C-S216A following doxycycline treatment was determined by phosphorylation of histone H3 (*pH3*) staining. The percentage of M-phase cells in vehicle-treated cells were set to 100, and the value of AMPK activator–treated cells was normalized to that of vehicle-treated cells. *Error bars*, S.D. of triplicate samples (*left*). Cell lysates were blotted with the indicated antibodies (*right*). *, *p* < 0.05; **, *p* < 0.01.

Our data on AMPK-dependent phosphorylation of Ser-216 on CDC25C suggested that AMPK suppresses the G_2_/M-phase transition through the phosphorylation and inhibition of CDC25C. CDC25C activates CDC2-cyclin B and promotes the G_2_/M-phase transition by antagonizing WEE1 (*i.e.* by dephosphorylating WEE1-dependent phosphorylation sites on CDC2-cyclin B) (42, 87, 88). We therefore reasoned that the abrogation of WEE1 or the abrogation of Ser-216 on CDC25C would relieve AMPK-dependent inhibition of the G_2_/M-phase transition (Fig. 6*A*). To test this, we first took a pharmacological approach, using MK1775, to block WEE1 activity in the presence of AMPK activators. MK1775 is a potent WEE1 inhibitor and allows us to acutely block WEE1 activity in cells synchronized at G_2_ phase without interfering with the cell-cycle synchronization process (89). Because a pharmacological inhibitor readily suppresses its target upon addition, we treated cells with MK1775 at the same time as we added AMPK inhibitors, ensuring a minimal perturbation by the compound on G_2_-phase progression (Fig. 6*B*). Supporting the idea that the inhibition of WEE1 may antagonize AMPK-mediated suppression on CDC25C, neither A7 nor AICAR treatment delayed mitosis entry in the presence of MK1775; however, A7 and AICAR treatment consistently reduced the percentage of cells in mitosis in the absence of MK1775 (Fig. S4, *A* and *B*). Next, we applied a genetic approach to knock down WEE1 by transfecting a WEE1-specific siRNA. To avoid perturbing the normal cell-cycle progression following a long-term WEE1 knockdown, we transfected cells with WEE1 siRNA when synchronized cells were released into S phase and collected samples 12 h later to examine the protein level of WEE1 and cell-cycle status (Fig. 6*B*). The protein level of mammalian WEE1 oscillates during the cell cycle and thus indicates a short half-life of WEE1 protein. This allows us to deplete WEE1 in a short time frame by siRNA (Fig. 6*C*) (90–92). Consistent with our data on MK1775, siRNA-mediated acute knockdown of WEE1 also partially relieved AMPK-mediated suppression of the G_2_/M transition (Fig. 6*C* and Fig. S4*C*). Last, we sought to genetically modulate the phosphorylation of Ser-216 on CDC25C and examine its effect on G_2_/M-phase transition in the context of AMPK activation. To minimize the perturbation of constitutive overexpression of CDC25C on normal cell-cycle progression, we established a cell line stably expressing a doxycycline-inducible CDC25C (S216A) mutant, which enables the acute induction of CDC25C (S216A) following doxycycline treatment when synchronized cells were released into S phase (Fig. 6*B*). We confirmed the acute induction of CDC25C (S216A) by immunoblotting and showed that such treatment partially relieved AMPK-mediated suppression of the G_2_/M-phase transition (Fig. 6*D* and Fig. S4*D*). Of note, neither the WEE1 siRNA nor the induction of CDC25C (S216A) in the context of AMPK activation caused additional stresses and induced cell death during the time frame of experiments (Fig. S5). Taken together, our results suggest that AMPK-mediated phosphorylation of Ser-216 on CDC25C represents a mechanism of cell-cycle checkpoint.

### WEE1 inhibitor synergizes with AMPK activators to induce cell death

**Figure 7.**
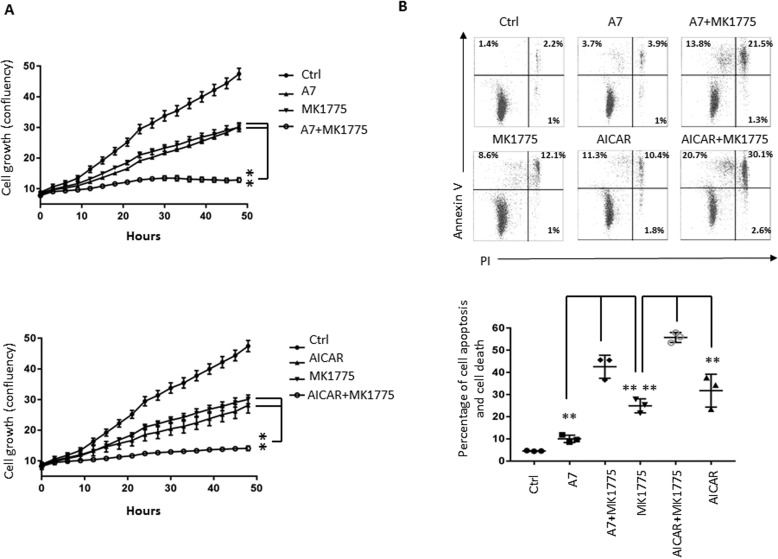
**Pharmacological ablation of G_2_ checkpoint synergizes with AMPK activators to induce cell death.** *A*, HeLa cells were treated with AMPK activators, MK1775, or a combination of the two, and the growth curve was determined by live cell imagine analysis (IncuCyte ZOOM^TM^). *Error bars*, S.D. of quadruplicate samples. *B*, HeLa cells were treated with AMPK activators, MK1775, or a combination for 48 h, and cell death was determined by staining with FITC-conjugated annexin V and PI followed by FACS analysis. The percentage of apoptotic and dead cells was determined from three experiments. *Bars*, mean ± S.D. **, *p* < 0.01.

AMPK is a central sensor of cellular energy status and therefore plays a key role in maintaining metabolic and bioenergetic homeostasis (26, 93). We envisioned that AMPK-mediated suppression on G_2_/M-phase transition may represent a metabolic checkpoint that ensures the coordination of sequential cell-cycle transitions with metabolic status. As such, abrogation of the checkpoint may reduce the ability of cells to survive. To test this idea, we treated cells with AMPK activator, WEE1 inhibitor, or a combination of these two and monitored the cell growth curve. Whereas single-agent treatment demonstrated a moderate ability to suppress cell growth, either A7 or AICAR in combination with MK1175 led to a synergistic effect on suppressing cell growth (Fig. 7*A*). Moreover, the growth suppression correlated with cell death induction, as measured by cell surface staining of annexin V and cellular uptake of PI, following the treatment with these reagents (Fig. 7*B*). Our studies suggest that abrogation of AMPK-dependent G_2_ checkpoint response induces cell death and may represent an attractive cancer therapeutic strategy.

### Metabolic stress suppresses the G_2_/M transition partially through AMPK

**Figure 8.**
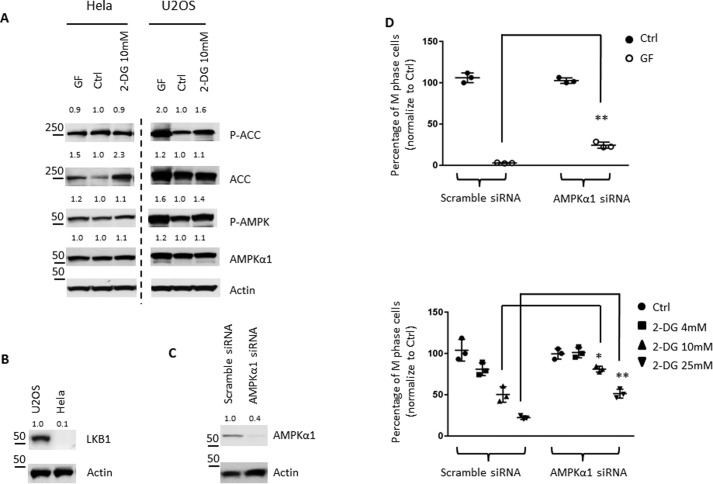
**Metabolic stress suppresses G_2_/M transition partially through AMPK.** *A*, U2OS and HeLa cells were cultured in glucose-free medium or treated with 10 mm 2DG for 1 h. The level of the indicated proteins in cells was determined by immunoblotting. *B*, U2OS and HeLa cells were collected, and the level of the indicated proteins was determined by immunoblot. *C*, U2OS cells were transfected with scrambled siRNA or AMPKα1 siRNA for 48 h. The level of the indicated proteins in cells was determined by immunoblot. *D*, U2OS cells were transfected with scrambled siRNA or AMPKα1 siRNA for 48 h and then incubated in control or glucose-free medium (*GF*, *top*) or treated with a different concentration of 2DG (*bottom*) along with 100 ng/ml nocodazole for 14 h. The percentage of M-phase cells was determined by the phosphorylation of histone H3 (*pH3*) staining. The percentage of M-phase cells in control medium was set to 100, and the value of cells in GF medium or 2DG-treated cells was normalized to their control. *Error bars*, S.D. of triplicate samples.

We next sought to assess the impact of metabolic stress on AMPK and cell-cycle G_2_/M transition. We first applied glucose-free conditional medium and hexokinase inhibitor, 2-deoxyglucose (2DG), to induce an acute metabolic stress and assess the activation of AMPK. Given that the average duration of G_2_ phase is between 2 and 5 h, we reasoned that the acute condition (1 h) is more relevant to assess the impact of metabolic stress on cell-cycle G_2_/M transition. Under this condition, we found that U2OS cells but not HeLa cells respond to acute metabolic stress by enhancing AMPK activity, as indicated by the increase of AMPK autophosphorylation and phosphorylation of ACC (Fig. 8*A*). Previous studies have shown that HeLa cells are deficient in liver kinase B1 (LKB1), an AMPK-activating kinase (94), and we have also confirmed LKB1 deficiency in HeLa cells by Western blotting (Fig. 8*B*). LKB1 deficiency probably renders HeLa cells resistant to metabolic stress-induced activation of AMPK because AMP/ADP promotes the conformational change of AMPK that will facilitate the phosphorylation by LKB1 and consequentially enhance AMPK activity (95, 96). As such, we chose U2OS cells to further assess the impact of metabolic stress on AMPK and on the G_2_/M transition. For this, we applied siRNA to transiently knock down AMPKα1 in U2OS cells (Fig. 8*C*). Both the procedure of transient transfection and the time frame (48 h) required to achieve an efficient knockdown of AMPKα1 interfered with the standard protocol for double thymidine synchronization. We therefore chose to treat cells with the indicated siRNA for 48 h and then applied nocodazole, to hold cells in mitosis, with or without metabolic stress. As Fig. 8*D* shows, metabolic stress imposed by either glucose starvation or glycolysis inhibitor 2DG significantly suppressed the percentage of cells entering into mitosis following nocodazole treatment. Importantly, AMPKα1 knockdown partially relieved metabolic stress-mediated suppression on the G_2_/M-phase transition (Fig. 8*D*). Collectively, our results suggested that acute metabolic stress (nutrient starvation or metabolic inhibitor) activates AMPK and suppresses the G_2_/M-phase transition.

Cell-cycle checkpoints are critical surveillance mechanisms that monitor the integrity and fidelity of genome replication and separation and thus ensure the order and timely execution of cell-cycle transitions (97–99). In addition, mammalian cells have evolved to rapidly respond to changes of internal metabolic status and external nutrient levels by engaging a mechanism of adaptation, which requires robust molecular machineries that sense metabolic signals. The consequences of such adaptation include not only metabolic rewiring but also commitments on cell-cycle progression, cell death, and many other basic or specialized cellular functions (100–102). Previous studies have shown that AMPK is crucial for the survival of cells by acting on an array of signaling and biochemical pathways to rapidly restore the energy status upon a variety of metabolic stresses (25, 26). Our studies implicate AMPK as a critical signaling node that interconnects with cell-cycle machinery through a direct phosphorylation and suppression of CDC25C. This allows cells to constantly inspect the metabolic status, halt the cell-cycle progression upon perturbations, and therefore ensure the cellular metabolic fitness and homeostasis of the progenies. Our studies further suggest that phosphorylation of Ser-216 in CDC25C is not only an evolutionarily conserved mechanism in normal cell-cycle regulation, but also a shared G_2_-phase checkpoint mechanism in mammalian cells in response to both DNA damage and metabolic stress (51, 53, 54, 61, 62). These findings together support the idea that some key elements of the cellular stress response pathways are shared among different stresses and are conserved across many species (103, 104). In multicellular eukaryotes, programmed cell death (apoptosis) occurs when the dose of stress exceeds tolerance limits or when adaptive pro-survival checkpoint mechanisms are eliminated (105–107). MK1775 has displayed anti-tumor activities in various preclinical models and has been evaluated in clinical trials as monotherapy and adjuvant therapy (108, 109). Our studies therefore suggest that abrogation of the metabolic checkpoint on G_2_/M-phase transition by WEE1 inhibitor may lead to premature mitosis entry and consequent cell death and therefore holds promise for therapeutically targeting cancer (110–112).

## Experimental procedures

### Cell culture and reagents

HeLa (human cervical cancer cells, ATCC) and U2OS (human osteosarcoma cells, ATCC) were grown at 37 ºC/5% CO_2_ in Dulbecco's modified Eagle's medium (DMEM; Corning Cellgro, Thermo Fisher Scientific, Grand Island, NY) supplemented with 10% fetal bovine serum (Gibco-Invitrogen, Carlsbad, CA) and 1% penicillin-streptomycin (Corning). Cell line identity was authenticated by short tandem repeat analysis. Glucose-free DMEM was supplemented with 10% (v/v) heat-inactivated dialyzed fetal bovine serum, which was made dialyzing against 100 volumes of distilled water (five changes in 3 days) using Slide-ALyzer^TM^ G_2_ dialysis cassettes with cut-through MW size 2K (Thermo Fisher Scientific) at 4 °C. AICAR, A769662, rapamycin, torin, metformin, phenformin, 2-deoxy-d-glucose, and nocodazole were purchased from Caymen Chemical (Ann Arbor, MI). MK1775 was purchased from MedKoo Biosciences (Morrisville, NC).

### siRNA transfection

The siRNA oligonucleotides corresponding to human WEE1 and AMPKα1 were purchased from Fisher. siRNA oligonucleotides (20 nm) were transfected into cells using Lipofectamine RNAiMAX reagent (Invitrogen). After 12 h of transfection (transfection when cells were released from the second thymidine block) or after 48 h of transfection (transfection in non-synchronized cells), immunoblots were carried out to examine the knockdown of targeted proteins.

### RNA isolation, reverse transcription, and quantitative PCR

Total RNA was isolated using an RNA extraction kit (Zymo Research, Irvine, CA) and was reverse-transcribed using random hexamer and Moloney murine leukemia virus reverse transcriptase (Invitrogen). SYBR Green-based quantitative RT-PCR for specific genes was performed using the Applied Biosystems real-time PCR system. Samples for each experimental condition were run in triplicate and were normalized to β_2_-microglobulin to determine relative expression levels. Primer sequences (Table S2) were obtained from the Primer Bank (113).

### Protein extraction and Western blot analysis

Cells were harvested, lysed, and sonicated at 4 °C in a lysis buffer (50 mm Tris-HCl, pH 7.4, 150 mm NaCl, 0.5% SDS, 5 mm sodium pyrophosphate, protease, and phosphatase inhibitor tablet). Cell lysates were centrifuged at 13,000 × *g* for 15 min, and the supernatant was recovered. The protein concentrations were determined using the Pierce^TM^ BCA protein assay kit (Thermo Fisher Scientific). After 5 min boiling in 4× NuPAGE® LDS sample buffer with 10× reducing solution (Thermo Fisher Scientific), the proteins were separated by NuPAGE 4–12% protein gels (Thermo Fisher Scientific), transferred to polyvinylidene difluoride membranes by using the iBlot gel transfer device (Thermo Fisher Scientific), and probed with the appropriate primary antibodies (Table S1). Membrane-bound primary antibodies were detected using secondary antibodies conjugated with horseradish peroxidase. Immunoblots were developed on films using the enhanced chemiluminescence technique. Density of each band was analyzed by ImageJ software, and all values were normalized with actin. All image data are representative of at least two independent experiments.

### Synchronization and cell-cycle analysis

HeLa cells were synchronized to the G_1_/S border by double thymidine blockage and released into fresh medium for 7 h until they reached G_2_ phase. In some experiments, HeLa cells were synchronized in mitosis by a shake-off after 14 h of incubation in 100 ng/ml nocodazole, washed, and allowed to transition to G_1_ phase. U2OS cells were synchronized to M phase by 14 h of incubation in 100 ng/ml nocodazole.

BrdU incorporation was used to evaluate the amount of S-phase cells. In short, after labeling of cells with BrdU (Sigma-Aldrich), cells were fixed in ice-cold ethanol, treated with HCl to denature the DNA, and stained with PI and anti-BrdU antibody. Mitotic cells were examined by intracellular staining of phospho-histone H3 using a hypotonic buffer protocol described previously (114). Cell-cycle analysis was done with a flow cytometer (NovoCyte, ACEA Biosciences, San Diego, CA), and FlowJo version 10 was used to analyze the flow cytometry data.

### In vitro phosphorylation

The bacterial expression vectors for GST-tagged CDC25 fragments were generated by PCR amplification of the encoding human CDC25C cDNA, followed by subcloning of the cDNA into pGEX-4T3 vector. Activated recombinant ERK (p42 MAPK) was prepared by incubating MBP-MAPK with constitutively activated MBP-MKK1 (115). GST- and MBP-tagged recombinant proteins were expressed in bacteria and purified as described previously (49, 115, 116). AMP kinase was purchased from Millipore (Burlington, MA). The kinase reactions were performed in 10 μl of reaction mixture (1 μl of 10× kinase buffer and 0.25 mm ATP), AMPK (1 unit), and 1 μg of the indicated substrates for 30 min at 30 °C. The reaction mixtures were boiled in SDS sample buffer and subjected to SDS-PAGE analysis and a Western blot assay.

### Metabolic activity analysis

The radioactive tracers are described in Table S3. The activity of various metabolic pathways was determined by the rate of detritiation or ^14^CO_2_ released from radioactive tracers, as described previously (117, 118). Specifically, glycolysis and fatty acid β-oxidation were determined by measuring the detritiation of [5-^3^H]glucose (119, 120) or by measuring the detritiation of [9,10-^3^H]palmitic acid (121), respectively. In brief, 1 million cells were suspended in 0.5 ml of fresh medium. The experiment was initiated by adding 1 μCi of radioactive tracer, and 2 h later, medium was transferred to a 1.5-ml microcentrifuge tube containing 50 μl of 5 n HCl. The microcentrifuge tubes were then placed in 20-ml scintillation vials containing 0.5 ml of water with the vials capped and sealed. ^3^H_2_O was separated from other radiolabeled metabolites by evaporation diffusion for 24 h at room temperature. A cell-free sample containing 1 μCi of radioactive tracer was included as a background control.

Glutaminolysis, TCA cycle (oxidation of pyruvate), and glucose oxidation flux through the PPP were determined by the rate of ^14^CO_2_ released from [U-^14^C]glutamine (122), the rate of ^14^CO_2_ released from [2-^14^C]pyruvate (123), and the rate of ^14^CO_2_ released from [1-^14^C]glucose with some modifications (123). Whereas the difference in the rate of ^14^CO_2_ released from [1-^14^C]glucose and [6-^14^C]glucose was used to determine the PPP activity, we consistently found that the ^14^CO_2_ production from [6-^14^C]glucose was close to the background in cells. PPP activity was therefore determined as the rate of ^14^CO_2_ released from [1-^14^C]glucose. In brief, 3 million cells were suspended in 0.5 ml of fresh medium. To facilitate the collection of ^14^CO_2_, cells were dispensed into 7-ml glass vials (TS-13028, Thermo Fisher Scientific) with a PCR tube containing 50 μl of 0.2 m KOH glued on the sidewall. After adding 0.5 μCi of radioactive tracer, the vials were capped using a screw cap with a rubber septum (TS-12713, Thermo Fisher Scientific). The assay was stopped 2 h later by injection of 100 μl of 5 n HCl, and the vials were kept at room temperature overnight to trap the ^14^CO_2_. The 50 μl of KOH in the PCR tube was then transferred to scintillation vials containing 10 ml of scintillation solution for counting. A cell-free sample containing 0.5 μCi of radioactive tracer was included as a background control.

### Phosphorylation of CDC25C in cells

HeLa cells were seeded at 4 × 10^5^ cells/35-mm dish and were transfected with 1.25 μg of WT- or AS-AMPKα2 and 1.25 μg of WT- or S216A-Myc-CDC25C using the Lipofectamine (Thermo Fisher Scientific). Forty-eight hours after transfection, cells were washed twice with serum-free DMEM and incubated for 2 h before stimulation with 300 μm A769662 for 20 min. Following stimulation, 200 μl of phosphorylation buffer (20 mm HEPES (pH 7.3), 100 mm KOAc, 5 mm NaOAc, 2 mm MgOAc_2_, 1 mm EGTA, 10 mm MgCl_2_, 0.5 mm DTT, 5 mm creatine phosphate, 57 μg/ml creatine kinase, 30 μg/ml digitonin, 5 mm GTP (Abcam), 0.1 mm ATP, 0.1 mm *N*^6^-(phenethyl) ATPγS (Abcam), 0.45 mm AMP (Abcam), 1× phosphatase inhibitor mixture I and II (Sigma), and 1× complete protease inhibitors (Sigma)) was added. After a 20-min incubation at room temperature, 200 μl of 2× radioimmune precipitation assay buffer (100 mm Tris, pH 8, 300 mm NaCl, 2% Nonidet P-40, 0.2% SDS, and 20 mm EDTA) with 2.5 mm *p*-nitrobenzyl mesylate (Abcam) and 5% DMSO was added, and cells were incubated for an additional 1 h at room temperature. The cell lysates were then subjected to immunoprecipitation using agarose beads coupled to Myc tag antibody.

### Constructs and generation of CDC25C-inducible stable cell lines

Plasmid pECE-AMPKα2 WT, M93G, and pEBG-AMPKα1 (1–312) were obtained from Addgene (Cambridge, MA) (82, 86). The pRetro-TRE3G vector (Clontech, Mountain View, CA) that expresses doxycycline-inducible CDC25C-S216A was generated by a recombination-based cloning method (In-Fusion Cloning Kits, Clontech) followed by site-directed mutagenesis (New England Biolabs, Ipswich, MA). Myc-tagged CDC25C-S216A expression plasmid was generated by subcloning pRetro-TRE3G-CDC25C-S216A into pCS2+MT plasmid.

The Amphopack 293 cells were transfected with the pRetro-TRE3G vector using Lipofectamine (Thermo Fisher Scientific) to produce retrovirus. HeLa cells that stably expressed reverse tetracycline-controlled transactivator (rtTA, Clontech) were infected with the retrovirus and treated with 2 μg/ml puromycin (Sigma) and maintained in puromycin-containing medium.

### Cell growth and cell death

Cell proliferations were measured by the IncuCyte cell proliferation assay. Cells were harvested by trypsinization, counted on a Countess automated cell counter (Invitrogen), and plated at 4000 cells/well on 96-well tissue culture plates in four replicates. Photomicrographs were taken every 3 h using an IncuCyte live cell imager (Essen Biosciences, Ann Arbor, MI), and confluence of the cultures was measured using IncuCyte software (Essen Biosciences) over 48 h in culture. For the cell death assay, cells were stained with annexin V–APC and PI and evaluated for apoptosis by flow cytometry according to the manufacturer's protocol (BD PharMingen, San Diego, CA).

### Statistical analysis

*p* values were calculated with Student's *t* test. *p* values < 0.05 were considered significant, with *p* values < 0.05 and *p* values < 0.01 indicated as with single and double asterisks, respectively.

## Author contributions

Y. S. data curation; Y. S. formal analysis; Y. S. validation; Y. S. and J. W. S. investigation; Y. S., J. W. S., and X. C. methodology; Y. S. and R. W. writing-original draft; Y. S., J. W. S., X. C., and R. W. writing-review and editing; R. W. conceptualization; R. W. supervision.

## Supplementary Material

Supporting Information

## Footnotes

2 The abbreviations used are:

AMPK: AMP-activated protein kinase
CDK: cyclin-dependent kinase
mTOR: mammalian target of rapamycin
AICAR: 5-aminoimidazole-4-carboxamide 1-β-d-ribofuranoside
PI: propidium iodide
BrdU: bromodeoxyuridine
PPP: pentose phosphate pathway
ERK: extracellular signal-regulated kinase
ATPγS: adenosine 5′-*O*-(thiotriphosphate)
AS: analog-specific
thioP: thiophosphate
2DG: 2-deoxyglucose
LKB1: liver kinase B1
DMEM: Dulbecco's modified Eagle's medium
GST: glutathione *S*-transferase
MBP: maltose-binding protein.

## References

[1] Robbins E., and Morrill G. A. (1969) Oxygen uptake during the HeLa cell life cycle and its correlation with macromolecular synthesis. J. Cell Biol. 43, 629–633 10.1083/jcb.43.3.629 5351410 PMC2107809

[2] Bendick C., Rasokat H., and Steigleder G. K. (1989) Azidothymidine-induced hyperpigmentation of skin and nails. Arch. Dermatol. 125, 1285–1286 10.1001/archderm.1989.01670210123028 2774609

[3] Buchakjian M. R., and Kornbluth S. (2010) The engine driving the ship: metabolic steering of cell proliferation and death. Nat. Rev. Mol. Cell Biol. 11, 715–727 10.1038/nrm2972 20861880

[4] Cai L., and Tu B. P. (2012) Driving the cell cycle through metabolism. Annu. Rev. Cell Dev. Biol. 28, 59–87 10.1146/annurev-cellbio-092910-154010 22578140

[5] Jorgensen P., and Tyers M. (2004) How cells coordinate growth and division. Curr. Biol. 14, R1014–1027 10.1016/j.cub.2004.11.027 15589139

[6] Lee I. H., and Finkel T. (2013) Metabolic regulation of the cell cycle. Curr. Opin. Cell Biol. 25, 724–729 10.1016/j.ceb.2013.07.002 23890700 PMC3836844

[7] Foster D. A., Yellen P., Xu L., and Saqcena M. (2010) Regulation of G_1_ cell cycle progression: distinguishing the restriction point from a nutrient-sensing cell growth checkpoint(s). Genes Cancer 1, 1124–1131 10.1177/1947601910392989 21779436 PMC3092273

[8] Hartwell L. H., Culotti J., Pringle J. R., and Reid B. J. (1974) Genetic control of the cell division cycle in yeast. Science 183, 46–51 10.1126/science.183.4120.46 4587263

[9] Ho A., and Dowdy S. F. (2002) Regulation of G_1_ cell-cycle progression by oncogenes and tumor suppressor genes. Curr. Opin. Genet. Dev. 12, 47–52 10.1016/S0959-437X(01)00263-5 11790554

[10] Pardee A. B. (1974) A restriction point for control of normal animal cell proliferation. Proc. Natl. Acad. Sci. U.S.A. 71, 1286–1290 10.1073/pnas.71.4.1286 4524638 PMC388211

[11] Sherr C. J. (1996) Cancer cell cycles. Science 274, 1672–1677 10.1126/science.274.5293.1672 8939849

[12] Almeida A., Bolaños J. P., and Moncada S. (2010) E3 ubiquitin ligase APC/C-Cdh1 accounts for the Warburg effect by linking glycolysis to cell proliferation. Proc. Natl. Acad. Sci. U.S.A. 107, 738–741 10.1073/pnas.0913668107 20080744 PMC2818939

[13] Birsoy K., Wang T., Chen W. W., Freinkman E., Abu-Remaileh M., and Sabatini D. M. (2015) An essential role of the mitochondrial electron transport chain in cell proliferation is to enable aspartate synthesis. Cell 162, 540–551 10.1016/j.cell.2015.07.016 26232224 PMC4522279

[14] Colombo S. L., Palacios-Callender M., Frakich N., De Leon J., Schmitt C. A., Boorn L., Davis N., and Moncada S. (2010) Anaphase-promoting complex/cyclosome-Cdh1 coordinates glycolysis and glutaminolysis with transition to S phase in human T lymphocytes. Proc. Natl. Acad. Sci. U.S.A. 107, 18868–18873 10.1073/pnas.1012362107 20921411 PMC2973876

[15] Sullivan L. B., Gui D. Y., Hosios A. M., Bush L. N., Freinkman E., and Vander Heiden M. G. (2015) Supporting aspartate biosynthesis is an essential function of respiration in proliferating cells. Cell 162, 552–563 10.1016/j.cell.2015.07.017 26232225 PMC4522278

[16] Ferrell J. E. Jr. (2013) Feedback loops and reciprocal regulation: recurring motifs in the systems biology of the cell cycle. Curr. Opin. Cell Biol. 25, 676–686 10.1016/j.ceb.2013.07.007 23927869 PMC3836843

[17] Mitchison J. M. (1972) The Biology of the Cell Cycle, pp. 192–200, Cambridge University Press, Cambridge, UK

[18] Pederson T. (2003) Historical review: an energy reservoir for mitosis, and its productive wake. Trends Biochem. Sci. 28, 125–129 10.1016/S0968-0004(03)00030-6 12633991

[19] Swann M. M. (1957) The control of cell division: a review. I. General mechanisms. Cancer Res. 17, 727–757 13460979

[20] Di Talia S., Skotheim J. M., Bean J. M., Siggia E. D., and Cross F. R. (2007) The effects of molecular noise and size control on variability in the budding yeast cell cycle. Nature 448, 947–951 10.1038/nature06072 17713537

[21] Ginzberg M. B., Kafri R., and Kirschner M. (2015) Cell biology: on being the right (cell) size. Science 348, 1245075 10.1126/science.1245075 25977557 PMC4533982

[22] Martin S. G., and Berthelot-Grosjean M. (2009) Polar gradients of the DYRK-family kinase Pom1 couple cell length with the cell cycle. Nature 459, 852–856 10.1038/nature08054 19474792

[23] Moseley J. B., Mayeux A., Paoletti A., and Nurse P. (2009) A spatial gradient coordinates cell size and mitotic entry in fission yeast. Nature 459, 857–860 10.1038/nature08074 19474789

[24] Tzur A., Kafri R., LeBleu V. S., Lahav G., and Kirschner M. W. (2009) Cell growth and size homeostasis in proliferating animal cells. Science 325, 167–171 10.1126/science.1174294 19589995 PMC2905160

[25] Hardie D. G., Ross F. A., and Hawley S. A. (2012) AMPK: a nutrient and energy sensor that maintains energy homeostasis. Nat. Rev. Mol. Cell Biol. 13, 251–262 10.1038/nrm3311 22436748 PMC5726489

[26] Herzig S., and Shaw R. J. (2018) AMPK: guardian of metabolism and mitochondrial homeostasis. Nat. Rev. Mol. Cell Biol. 19, 121–135 10.1038/nrm.2017.95 28974774 PMC5780224

[27] Igata M., Motoshima H., Tsuruzoe K., Kojima K., Matsumura T., Kondo T., Taguchi T., Nakamaru K., Yano M., Kukidome D., Matsumoto K., Toyonaga T., Asano T., Nishikawa T., and Araki E. (2005) Adenosine monophosphate-activated protein kinase suppresses vascular smooth muscle cell proliferation through the inhibition of cell cycle progression. Circ. Res. 97, 837–844 10.1161/01.RES.0000185823.73556.06 16151020

[28] Imamura K., Ogura T., Kishimoto A., Kaminishi M., and Esumi H. (2001) Cell cycle regulation via p53 phosphorylation by a 5′-AMP activated protein kinase activator, 5-aminoimidazole- 4-carboxamide-1-β-d-ribofuranoside, in a human hepatocellular carcinoma cell line. Biochem. Biophys. Res. Commun. 287, 562–567 10.1006/bbrc.2001.5627 11554766

[29] Liang J., Shao S. H., Xu Z. X., Hennessy B., Ding Z., Larrea M., Kondo S., Dumont D. J., Gutterman J. U., Walker C. L., Slingerland J. M., and Mills G. B. (2007) The energy sensing LKB1-AMPK pathway regulates p27(kip1) phosphorylation mediating the decision to enter autophagy or apoptosis. Nat. Cell Biol. 9, 218–224 10.1038/ncb1537 17237771

[30] Rattan R., Giri S., Singh A. K., and Singh I. (2005) 5-Aminoimidazole-4-carboxamide-1-β-d-ribofuranoside inhibits cancer cell proliferation *in vitro* and *in vivo* via AMP-activated protein kinase. J. Biol. Chem. 280, 39582–39593 10.1074/jbc.M507443200 16176927

[31] Ben-Sahra I., and Manning B. D. (2017) mTORC1 signaling and the metabolic control of cell growth. Curr. Opin. Cell Biol. 45, 72–82 10.1016/j.ceb.2017.02.012 28411448 PMC5545101

[32] Fingar D. C., Richardson C. J., Tee A. R., Cheatham L., Tsou C., and Blenis J. (2004) mTOR controls cell cycle progression through its cell growth effectors S6K1 and 4E-BP1/eukaryotic translation initiation factor 4E. Mol. Cell. Biol. 24, 200–216 10.1128/MCB.24.1.200-216.2004 14673156 PMC303352

[33] Gwinn D. M., Shackelford D. B., Egan D. F., Mihaylova M. M., Mery A., Vasquez D. S., Turk B. E., and Shaw R. J. (2008) AMPK phosphorylation of raptor mediates a metabolic checkpoint. Mol. Cell 30, 214–226 10.1016/j.molcel.2008.03.003 18439900 PMC2674027

[34] Inoki K., Zhu T., and Guan K. L. (2003) TSC2 mediates cellular energy response to control cell growth and survival. Cell 115, 577–590 10.1016/S0092-8674(03)00929-2 14651849

[35] Saxton R. A., and Sabatini D. M. (2017) mTOR signaling in growth, metabolism, and disease. Cell 169, 361–371 10.1016/j.cell.2017.03.035 28388417

[36] Shaw R. J., Bardeesy N., Manning B. D., Lopez L., Kosmatka M., DePinho R. A., and Cantley L. C. (2004) The LKB1 tumor suppressor negatively regulates mTOR signaling. Cancer Cell 6, 91–99 10.1016/j.ccr.2004.06.007 15261145

[37] Dunphy W. G., Brizuela L., Beach D., and Newport J. (1988) The *Xenopus* cdc2 protein is a component of MPF, a cytoplasmic regulator of mitosis. Cell 54, 423–431 10.1016/0092-8674(88)90205-X 3293802

[38] Gautier J., Norbury C., Lohka M., Nurse P., and Maller J. (1988) Purified maturation-promoting factor contains the product of a *Xenopus* homolog of the fission yeast cell cycle control gene cdc2+. Cell 54, 433–439 10.1016/0092-8674(88)90206-1 3293803

[39] Morgan D. O. (2006) The Cell Cycle: Principles of Control, 1st Ed., pp. 90–102, New Science Press, London, UK

[40] Nurse P. (1975) Genetic control of cell size at cell division in yeast. Nature 256, 547–551 10.1038/256547a0 1165770

[41] King R. W., Jackson P. K., and Kirschner M. W. (1994) Mitosis in transition. Cell 79, 563–571 10.1016/0092-8674(94)90542-8 7954823

[42] Perdiguero E., and Nebreda A. R. (2004) Regulation of Cdc25C activity during the meiotic G_2_/M transition. Cell Cycle 3, 733–737 15136768

[43] Crenshaw D. G., Yang J., Means A. R., and Kornbluth S. (1998) The mitotic peptidyl-prolyl isomerase, Pin1, interacts with Cdc25 and Plx1. EMBO J. 17, 1315–1327 10.1093/emboj/17.5.1315 9482729 PMC1170480

[44] Izumi T., Walker D. H., and Maller J. L. (1992) Periodic changes in phosphorylation of the *Xenopus* cdc25 phosphatase regulate its activity. Mol. Biol. Cell 3, 927–939 10.1091/mbc.3.8.927 1392080 PMC275649

[45] Kumagai A., and Dunphy W. G. (1992) Regulation of the cdc25 protein during the cell cycle in *Xenopus* extracts. Cell 70, 139–151 10.1016/0092-8674(92)90540-S 1623517

[46] Kumagai A., and Dunphy W. G. (1996) Purification and molecular cloning of Plx1, a Cdc25-regulatory kinase from *Xenopus* egg extracts. Science 273, 1377–1380 10.1126/science.273.5280.1377 8703070

[47] Perdiguero E., Pillaire M. J., Bodart J. F., Hennersdorf F., Frödin M., Duesbery N. S., Alonso G., and Nebreda A. R. (2003) Xp38γ/SAPK3 promotes meiotic G_2_/M transition in *Xenopus* oocytes and activates Cdc25C. EMBO J. 22, 5746–5756 10.1093/emboj/cdg559 14592973 PMC275416

[48] Stukenberg P. T., and Kirschner M. W. (2001) Pin1 acts catalytically to promote a conformational change in Cdc25. Mol. Cell 7, 1071–1083 10.1016/S1097-2765(01)00245-3 11389853

[49] Wang R., He G., Nelman-Gonzalez M., Ashorn C. L., Gallick G. E., Stukenberg P. T., Kirschner M. W., and Kuang J. (2007) Regulation of Cdc25C by ERK-MAP kinases during the G_2_/M transition. Cell 128, 1119–1132 10.1016/j.cell.2006.11.053 17382881

[50] Wang R., Jung S. Y., Wu C. F., Qin J., Kobayashi R., Gallick G. E., and Kuang J. (2010) Direct roles of the signaling kinase RSK2 in Cdc25C activation during *Xenopus* oocyte maturation. Proc. Natl. Acad. Sci. U.S.A. 107, 19885–19890 10.1073/pnas.1003528107 21041626 PMC2993414

[51] Duckworth B. C., Weaver J. S., and Ruderman J. V. (2002) G_2_ arrest in *Xenopus* oocytes depends on phosphorylation of cdc25 by protein kinase A. Proc. Natl. Acad. Sci. U.S.A. 99, 16794–16799 10.1073/pnas.222661299 12477927 PMC139223

[52] Margolis S. S., Perry J. A., Forester C. M., Nutt L. K., Guo Y., Jardim M. J., Thomenius M. J., Freel C. D., Darbandi R., Ahn J. H., Arroyo J. D., Wang X. F., Shenolikar S., Nairn A. C., Dunphy W. G., et al (2006) Role for the PP2A/B56δ phosphatase in regulating 14-3-3 release from Cdc25 to control mitosis. Cell 127, 759–773 10.1016/j.cell.2006.10.035 17110335 PMC2789796

[53] Margolis S. S., Walsh S., Weiser D. C., Yoshida M., Shenolikar S., and Kornbluth S. (2003) PP1 control of M phase entry exerted through 14-3-3-regulated Cdc25 dephosphorylation. EMBO J. 22, 5734–5745 10.1093/emboj/cdg545 14592972 PMC275402

[54] Sanchez Y., Wong C., Thoma R. S., Richman R., Wu Z., Piwnica-Worms H., and Elledge S. J. (1997) Conservation of the Chk1 checkpoint pathway in mammals: linkage of DNA damage to Cdk regulation through Cdc25. Science 277, 1497–1501 10.1126/science.277.5331.1497 9278511

[55] Takizawa C. G., and Morgan D. O. (2000) Control of mitosis by changes in the subcellular location of cyclin-B1-Cdk1 and Cdc25C. Curr. Opin. Cell Biol. 12, 658–665 10.1016/S0955-0674(00)00149-6 11063929

[56] Ferrell J. E. Jr., and Ha S. H. (2014) Ultrasensitivity part II: multisite phosphorylation, stoichiometric inhibitors, and positive feedback. Trends Biochem. Sci. 39, 556–569 10.1016/j.tibs.2014.09.003 25440716 PMC4435807

[57] Boutros R., Lobjois V., and Ducommun B. (2007) CDC25 phosphatases in cancer cells: key players? Good targets? Nat. Rev. Cancer 7, 495–507 10.1038/nrc2169 17568790

[58] Donzelli M., and Draetta G. F. (2003) Regulating mammalian checkpoints through Cdc25 inactivation. EMBO Rep. 4, 671–677 10.1038/sj.embor.embor887 12835754 PMC1326326

[59] Karlsson-Rosenthal C., and Millar J. B. (2006) Cdc25: mechanisms of checkpoint inhibition and recovery. Trends Cell Biol. 16, 285–292 10.1016/j.tcb.2006.04.002 16682204

[60] Reinhardt H. C., and Yaffe M. B. (2009) Kinases that control the cell cycle in response to DNA damage: Chk1, Chk2, and MK2. Curr. Opin. Cell Biol. 21, 245–255 10.1016/j.ceb.2009.01.018 19230643 PMC2699687

[61] Furnari B., Rhind N., and Russell P. (1997) Cdc25 mitotic inducer targeted by chk1 DNA damage checkpoint kinase. Science 277, 1495–1497 10.1126/science.277.5331.1495 9278510

[62] Peng C. Y., Graves P. R., Thoma R. S., Wu Z., Shaw A. S., and Piwnica-Worms H. (1997) Mitotic and G_2_ checkpoint control: regulation of 14-3-3 protein binding by phosphorylation of Cdc25C on serine-216. Science 277, 1501–1505 10.1126/science.277.5331.1501 9278512

[63] Corton J. M., Gillespie J. G., Hawley S. A., and Hardie D. G. (1995) 5-Aminoimidazole-4-carboxamide ribonucleoside: a specific method for activating AMP-activated protein kinase in intact cells? Eur. J. Biochem. 229, 558–565 10.1111/j.1432-1033.1995.tb20498.x 7744080

[64] Day P., Sharff A., Parra L., Cleasby A., Williams M., Hörer S., Nar H., Redemann N., Tickle I., and Yon J. (2007) Structure of a CBS-domain pair from the regulatory γ1 subunit of human AMPK in complex with AMP and ZMP. Acta Crystallogr. D Biol. Crystallogr. 63, 587–596 10.1107/S0907444907009110 17452784

[65] Grahame Hardie D. (2016) Regulation of AMP-activated protein kinase by natural and synthetic activators. Acta Pharm. Sin. B 6, 1–19 10.1016/j.apsb.2015.06.002 26904394 PMC4724661

[66] Sanders M. J., Ali Z. S., Hegarty B. D., Heath R., Snowden M. A., and Carling D. (2007) Defining the mechanism of activation of AMP-activated protein kinase by the small molecule A-769662, a member of the thienopyridone family. J. Biol. Chem. 282, 32539–32548 10.1074/jbc.M706543200 17728241

[67] Foretz M., Guigas B., Bertrand L., Pollak M., and Viollet B. (2014) Metformin: from mechanisms of action to therapies. Cell Metab. 20, 953–966 10.1016/j.cmet.2014.09.018 25456737

[68] Branzei D., and Foiani M. (2008) Regulation of DNA repair throughout the cell cycle. Nat. Rev. Mol. Cell Biol. 9, 297–308 10.1038/nrm2351 18285803

[69] Hustedt N., and Durocher D. (2016) The control of DNA repair by the cell cycle. Nat. Cell Biol. 19, 1–9 28008184 10.1038/ncb3452

[70] Langerak P., and Russell P. (2011) Regulatory networks integrating cell cycle control with DNA damage checkpoints and double-strand break repair. Phil. Trans. R. Soc. Lond. B Biol. Sci. 366, 3562–3571 10.1098/rstb.2011.0070 22084383 PMC3203453

[71] Zhou B. B., and Elledge S. J. (2000) The DNA damage response: putting checkpoints in perspective. Nature 408, 433–439 10.1038/35044005 11100718

[72] Giglia-Mari G., Zotter A., and Vermeulen W. (2011) DNA damage response. Cold Spring Harb. Perspect. Biol. 3, a000745 20980439 10.1101/cshperspect.a000745 PMC3003462

[73] Jones R. G., Plas D. R., Kubek S., Buzzai M., Mu J., Xu Y., Birnbaum M. J., and Thompson C. B. (2005) AMP-activated protein kinase induces a p53-dependent metabolic checkpoint. Mol. Cell 18, 283–293 10.1016/j.molcel.2005.03.027 15866171

[74] Taylor W. R., and Stark G. R. (2001) Regulation of the G_2_/M transition by p53. Oncogene 20, 1803–1815 10.1038/sj.onc.1204252 11313928

[75] Vousden K. H., and Lu X. (2002) Live or let die: the cell's response to p53. Nat. Rev. Cancer 2, 594–604 10.1038/nrc864 12154352

[76] Hochegger H., Takeda S., and Hunt T. (2008) Cyclin-dependent kinases and cell-cycle transitions: does one fit all? Nat. Rev. Mol. Cell Biol. 9, 910–916 10.1038/nrm2510 18813291

[77] Malumbres M., and Barbacid M. (2009) Cell cycle, CDKs and cancer: a changing paradigm. Nat. Rev. Cancer 9, 153–166 10.1038/nrc2602 19238148

[78] Matlashewski G., Banks L., Pim D., and Crawford L. (1986) Analysis of human p53 proteins and mRNA levels in normal and transformed cells. Eur. J. Biochem. 154, 665–672 10.1111/j.1432-1033.1986.tb09449.x 2419131

[79] May E., Jenkins J. R., and May P. (1991) Endogenous HeLa p53 proteins are easily detected in HeLa cells transfected with mouse deletion mutant p53 gene. Oncogene 6, 1363–1365 1886712

[80] Scott J. W., Norman D. G., Hawley S. A., Kontogiannis L., and Hardie D. G. (2002) Protein kinase substrate recognition studied using the recombinant catalytic domain of AMP-activated protein kinase and a model substrate. J. Mol. Biol. 317, 309–323 10.1006/jmbi.2001.5316 11902845

[81] Yaffe M. B., Leparc G. G., Lai J., Obata T., Volinia S., and Cantley L. C. (2001) A motif-based profile scanning approach for genome-wide prediction of signaling pathways. Nat. Biotechnol. 19, 348–353 10.1038/86737 11283593

[82] Egan D. F., Shackelford D. B., Mihaylova M. M., Gelino S., Kohnz R. A., Mair W., Vasquez D. S., Joshi A., Gwinn D. M., Taylor R., Asara J. M., Fitzpatrick J., Dillin A., Viollet B., Kundu M., et al (2011) Phosphorylation of ULK1 (hATG1) by AMP-activated protein kinase connects energy sensing to mitophagy. Science 331, 456–461 10.1126/science.1196371 21205641 PMC3030664

[83] Alaimo P. J., Shogren-Knaak M. A., and Shokat K. M. (2001) Chemical genetic approaches for the elucidation of signaling pathways. Curr. Opin. Chem. Biol. 5, 360–367 10.1016/S1367-5931(00)00215-5 11470597

[84] Allen J. J., Lazerwith S. E., and Shokat K. M. (2005) Bio-orthogonal affinity purification of direct kinase substrates. J. Am. Chem. Soc. 127, 5288–5289 10.1021/ja050727t 15826144 PMC2943827

[85] Allen J. J., Li M., Brinkworth C. S., Paulson J. L., Wang D., Hübner A., Chou W. H., Davis R. J., Burlingame A. L., Messing R. O., Katayama C. D., Hedrick S. M., and Shokat K. M. (2007) A semisynthetic epitope for kinase substrates. Nat. Methods 4, 511–516 10.1038/nmeth1048 17486086 PMC2932705

[86] Banko M. R., Allen J. J., Schaffer B. E., Wilker E. W., Tsou P., White J. L., Villén J., Wang B., Kim S. R., Sakamoto K., Gygi S. P., Cantley L. C., Yaffe M. B., Shokat K. M., and Brunet A. (2011) Chemical genetic screen for AMPKα2 substrates uncovers a network of proteins involved in mitosis. Mol. Cell 44, 878–892 10.1016/j.molcel.2011.11.005 22137581 PMC3246132

[87] Boutros R., Dozier C., and Ducommun B. (2006) The when and wheres of CDC25 phosphatases. Curr. Opin. Cell Biol. 18, 185–191 10.1016/j.ceb.2006.02.003 16488126

[88] Perry J. A., and Kornbluth S. (2007) Cdc25 and Wee1: analogous opposites? Cell Div. 2, 12 10.1186/1747-1028-2-12 17480229 PMC1868713

[89] Hirai H., Iwasawa Y., Okada M., Arai T., Nishibata T., Kobayashi M., Kimura T., Kaneko N., Ohtani J., Yamanaka K., Itadani H., Takahashi-Suzuki I., Fukasawa K., Oki H., Nambu T., et al (2009) Small-molecule inhibition of Wee1 kinase by MK-1775 selectively sensitizes p53-deficient tumor cells to DNA-damaging agents. Mol. Cancer Ther. 8, 2992–3000 10.1158/1535-7163.MCT-09-0463 19887545

[90] McGowan C. H., and Russell P. (1995) Cell cycle regulation of human WEE1. EMBO J. 14, 2166–2175 7774574 10.1002/j.1460-2075.1995.tb07210.x PMC398322

[91] Wang Y., Jacobs C., Hook K. E., Duan H., Booher R. N., and Sun Y. (2000) Binding of 14-3-3β to the carboxyl terminus of Wee1 increases Wee1 stability, kinase activity, and G_2_-M cell population. Cell Growth Differ. 11, 211–219 10775038

[92] Watanabe N., Broome M., and Hunter T. (1995) Regulation of the human WEE1Hu CDK tyrosine 15-kinase during the cell cycle. EMBO J. 14, 1878–1891 7743995 10.1002/j.1460-2075.1995.tb07180.x PMC398287

[93] Hardie D. G., Schaffer B. E., and Brunet A. (2016) AMPK: an energy-sensing pathway with multiple inputs and outputs. Trends Cell Biol. 26, 190–201 10.1016/j.tcb.2015.10.013 26616193 PMC5881568

[94] Hurley R. L., Anderson K. A., Franzone J. M., Kemp B. E., Means A. R., and Witters L. A. (2005) The Ca^2+^/calmodulin-dependent protein kinase kinases are AMP-activated protein kinase kinases. J. Biol. Chem. 280, 29060–29066 10.1074/jbc.M503824200 15980064

[95] Hardie D. G., and Alessi D. R. (2013) LKB1 and AMPK and the cancer-metabolism link: ten years after. BMC Biol. 11, 36 10.1186/1741-7007-11-36 23587167 PMC3626889

[96] Shackelford D. B., and Shaw R. J. (2009) The LKB1-AMPK pathway: metabolism and growth control in tumour suppression. Nat. Rev. Cancer 9, 563–575 10.1038/nrc2676 19629071 PMC2756045

[97] Hartwell L. H., and Weinert T. A. (1989) Checkpoints: controls that ensure the order of cell cycle events. Science 246, 629–634 10.1126/science.2683079 2683079

[98] Kastan M. B., and Bartek J. (2004) Cell-cycle checkpoints and cancer. Nature 432, 316–323 10.1038/nature03097 15549093

[99] Weinert T. A., and Hartwell L. H. (1988) The RAD9 gene controls the cell cycle response to DNA damage in *Saccharomyces cerevisiae*. Science 241, 317–322 10.1126/science.3291120 3291120

[100] Hand S. C., and Hardewig I. (1996) Downregulation of cellular metabolism during environmental stress: mechanisms and implications. Annu. Rev. Physiol. 58, 539–563 10.1146/annurev.ph.58.030196.002543 8815808

[101] Kültz D. (2003) Evolution of the cellular stress proteome: from monophyletic origin to ubiquitous function. J. Exp. Biol. 206, 3119–3124 10.1242/jeb.00549 12909693

[102] Wang R., and Green D. R. (2012) Metabolic checkpoints in activated T cells. Nat. Immunol. 13, 907–915 10.1038/ni.2386 22990888

[103] Kültz D. (2005) Molecular and evolutionary basis of the cellular stress response. Annu. Rev. Physiol. 67, 225–257 10.1146/annurev.physiol.67.040403.103635 15709958

[104] Hochachka P. W., and Somero G. N. (2002) Biochemical Adaptation: Mechanism and Process in Physiological Evolution, 3rd Ed., pp. 101–157, Oxford University Press, Oxford, UK

[105] Green D. R., and Fitzgerald P. (2016) Just so stories about the evolution of apoptosis. Curr. Biol. 26, R620–R627 10.1016/j.cub.2016.05.023 27404257 PMC4972582

[106] Kawabe T. (2004) G_2_ checkpoint abrogators as anticancer drugs. Mol. Cancer Ther. 3, 513–519 15078995

[107] Shapiro G. I., and Harper J. W. (1999) Anticancer drug targets: cell cycle and checkpoint control. J. Clin. Invest. 104, 1645–1653 10.1172/JCI9054 10606615 PMC409893

[108] Do K., Wilsker D., Ji J., Zlott J., Freshwater T., Kinders R. J., Collins J., Chen A. P., Doroshow J. H., and Kummar S. (2015) Phase I study of single-agent AZD1775 (MK-1775), a Wee1 kinase inhibitor, in patients with refractory solid tumors. J. Clin. Oncol. 33, 3409–3415 10.1200/JCO.2014.60.4009 25964244 PMC4606059

[109] Leijen S., van Geel R. M., Pavlick A. C., Tibes R., Rosen L., Razak A. R., Lam R., Demuth T., Rose S., Lee M. A., Freshwater T., Shumway S., Liang L. W., Oza A. M., Schellens J. H., and Shapiro G. I. (2016) Phase I study evaluating WEE1 inhibitor AZD1775 as monotherapy and in combination with gemcitabine, cisplatin, or carboplatin in patients with advanced solid tumors. J. Clin. Oncol. 34, 4371–4380 10.1200/JCO.2016.67.5991 27601554 PMC7845944

[110] Aarts M., Sharpe R., Garcia-Murillas I., Gevensleben H., Hurd M. S., Shumway S. D., Toniatti C., Ashworth A., and Turner N. C. (2012) Forced mitotic entry of S-phase cells as a therapeutic strategy induced by inhibition of WEE1. Cancer Disc. 2, 524–539 10.1158/2159-8290.CD-11-0320 22628408

[111] Mir S. E., De Witt Hamer P. C., Krawczyk P. M., Balaj L., Claes A., Niers J. M., Van Tilborg A. A., Zwinderman A. H., Geerts D., Kaspers G. J., Peter Vandertop W., Cloos J., Tannous B. A., Wesseling P., Aten J. A., et al (2010) *In silico* analysis of kinase expression identifies WEE1 as a gatekeeper against mitotic catastrophe in glioblastoma. Cancer Cell 18, 244–257 10.1016/j.ccr.2010.08.011 20832752 PMC3115571

[112] Mueller S., and Haas-Kogan D. A. (2015) WEE1 kinase as a target for cancer therapy. J. Clin. Oncol. 33, 3485–3487 10.1200/JCO.2015.62.2290 26215953

[113] Wang X., Spandidos A., Wang H., and Seed B. (2012) PrimerBank: a PCR primer database for quantitative gene expression analysis, 2012 update. Nucleic Acids Res. 40, D1144–D1149 10.1093/nar/gkr1013 22086960 PMC3245149

[114] Shen Y., Vignali P., and Wang R. (2017) Rapid profiling cell cycle by flow cytometry using concurrent staining of DNA and mitotic markers. Bio Protoc. 7, e2517 10.21769/BioProtoc.2517 28868333 PMC5580980

[115] Rouse J., Cohen P., Trigon S., Morange M., Alonso-Llamazares A., Zamanillo D., Hunt T., and Nebreda A. R. (1994) A novel kinase cascade triggered by stress and heat shock that stimulates MAPKAP kinase-2 and phosphorylation of the small heat shock proteins. Cell 78, 1027–1037 10.1016/0092-8674(94)90277-1 7923353

[116] Palmer A., Gavin A. C., and Nebreda A. R. (1998) A link between MAP kinase and p34(cdc2)/cyclin B during oocyte maturation: p90(rsk) phosphorylates and inactivates the p34(cdc2) inhibitory kinase Myt1. EMBO J. 17, 5037–5047 10.1093/emboj/17.17.5037 9724639 PMC1170831

[117] Liu L., Lu Y., Martinez J., Bi Y., Lian G., Wang T., Milasta S., Wang J., Yang M., Liu G., Green D. R., and Wang R. (2016) Proinflammatory signal suppresses proliferation and shifts macrophage metabolism from Myc-dependent to HIF1α-dependent. Proc. Natl. Acad. Sci. U.S.A. 113, 1564–1569 10.1073/pnas.1518000113 26811453 PMC4760828

[118] Wang R., Dillon C. P., Shi L. Z., Milasta S., Carter R., Finkelstein D., McCormick L. L., Fitzgerald P., Chi H., Munger J., and Green D. R. (2011) The transcription factor Myc controls metabolic reprogramming upon T lymphocyte activation. Immunity 35, 871–882 10.1016/j.immuni.2011.09.021 22195744 PMC3248798

[119] Goodwin G. W., Cohen D. M., and Taegtmeyer H. (2001) [5-^3^H]Glucose overestimates glycolytic flux in isolated working rat heart: role of the pentose phosphate pathway. Am. J. Physiol. Endocrinol. Metab. 280, E502–508 10.1152/ajpendo.2001.280.3.E502 11171606

[120] Neely J. R., Denton R. M., England P. J., and Randle P. J. (1972) The effects of increased heart work on the tricarboxylate cycle and its interactions with glycolysis in the perfused rat heart. Biochem. J. 128, 147–159 10.1042/bj1280147 5085551 PMC1173579

[121] Moon A., and Rhead W. J. (1987) Complementation analysis of fatty acid oxidation disorders. J. Clin. Invest. 79, 59–64 10.1172/JCI112808 3793932 PMC423985

[122] Brand K., Williams J. F., and Weidemann M. J. (1984) Glucose and glutamine metabolism in rat thymocytes. Biochem. J. 221, 471–475 10.1042/bj2210471 6332620 PMC1144061

[123] Willems H. L., de Kort T. F., Trijbels F. J., Monnens L. A., and Veerkamp J. H. (1978) Determination of pyruvate oxidation rate and citric acid cycle activity in intact human leukocytes and fibroblasts. Clin. Chem. 24, 200–203 627049

