# Supplementary material for: Phosphorylation of CDC25C by AMP-activated protein kinase mediates a metabolic checkpoint during cell-cycle G2/M-phase transition
Source: J Biol Chem. 2018 Feb 21;293(14):5185–99. doi: 10.1074/jbc.RA117.001379 (PMC5892595; doi:10.1074/jbc.RA117.001379)
Supplement: Supporting Information [file supp_293_14_5185__index.html]

Phosphorylation of Cdc25C by AMP-activated protein kinase mediates a metabolic checkpoint during cell cycle G2/M phase transition — AMPK phosphorylates Cdc25C and regulates mitosis entry — Phosphorylation of CDC25C by AMP-activated protein kinase mediates a metabolic checkpoint during cell-cycle G2/M-phase transition — AMPK phosphorylates CDC25C and regulates mitosis entry — Supporting Information 

# Phosphorylation of CDC25C by AMP-activated protein kinase mediates a metabolic checkpoint during cell-cycle G2/M-phase transition

## Supporting Information

- Supplementary Figure legend - Supplementary Figure legend
- Figure S1 - Figure S1
- Figure S2 - Figure S2
- Figure S3 - Figure S3
- Figure S4 - Figure S4
- Figure S5 - Figure S5
- Supplementary Table - Supplementary Table 1,2,3
