# Supplementary material for: Phosphorylation of CDC25C by AMP-activated protein kinase mediates a metabolic checkpoint during cell-cycle G2/M-phase transition
Source: J Biol Chem. 2018 Feb 21;293(14):5185–99. doi: 10.1074/jbc.RA117.001379 (PMC5892595; doi:10.1074/jbc.RA117.001379)
Supplement: Supporting Information [file supp_RA117.001379_134549_1_supp_66929_p3h98v.docx]

**Figure S1. Acute activation of AMPK at G2 phase does not induce cell death.**

HeLa cells were treated with AMPK activator and Nocodazole when cells are in G2 phase. Cells were then collected 12 hours after the second Thymidine release and stained with FITC-conjugated Annexin V and propidium iodide (PI) for cell death analysis by FACS. The percentage of apoptotic and dead cells was determined from three experiments. Bars represent mean ± SD.

**Figure S2.** **Pharmacological activation of AMPK at G2 phase does not the expression of p21 and p27.**

The level of p21 and p27 mRNA in Hela cells following indicated treatments were determined by qPCR. Cells were collected as described in Figure 4A. MRNA levels at T0 were set to 1. Error bars represent standard deviation from the mean of triplicate samples.

**Figure S3. AMPK phosphorylates Cdc25C at Serine 216 in vitro and in cells.**

(A) Immunoprecipitation of lysate from Hela cells transfected with wildtype or serine 216 to alanine mutant (S216A) Myc-Cdc25C using Myc antibody and detected with indicated antibodies.

(B) Hela cells were treated 2mM AICAR at G2 phase alone with Nocodazole. Cells were collected at different time points as Figure 4A and lysate were blotted by indicated antibodies (* Mitotic shift bands of Cdc25C).

**Figure S4. Acute induction of Cdc25C or suppression of Wee 1 partially reverses the effect of AMPK activation on G2/M phase transition.**

(A) The percentage of M phase cells in Hela cells treated with MK1775 (Wee 1 inhibitor) was determined by phosphorylation of histone H3 (pH3) staining. The percentage of M phase cells in vehicle treated cells were set to 100 and the value of AMPK activator treated cells were normalized to vehicle treated cells. Error bars represent standard deviation from the mean of triplicate samples.

(B) The representative flow cytometric dot plots of Figure S4A.

(C) The representative flow cytometric dot plots of Figure 6C.

(D) The representative flow cytometric dot plots of Figure 6D.

**Figure S5. Acute induction of Cdc25C or suppression of Wee 1 does not affect cell death.**

(A) Cells that followed similar treatment as described in Figure 6C were stained with FITC-conjugated Annexin V and propidium iodide (PI) and were analyzed by FACS. The percentage of apoptotic and dead cells was determined from three experiments. Bars represent mean ± SD.

(B) Cells followed similar treatment as described in Figure 6D were stained with FITC-conjugated Annexin V and propidium iodide (PI) and were analyzed by FACS. The percentage of apoptotic and dead cells was determined from three experiments. Bars represent mean ± SD.
