# Supplementary figures and images for: Phosphorylation of CDC25C by AMP-activated protein kinase mediates a metabolic checkpoint during cell-cycle G2/M-phase transition
Source: J Biol Chem. 2018 Feb 21;293(14):5185–99. doi: 10.1074/jbc.RA117.001379 (PMC5892595; doi:10.1074/jbc.RA117.001379)

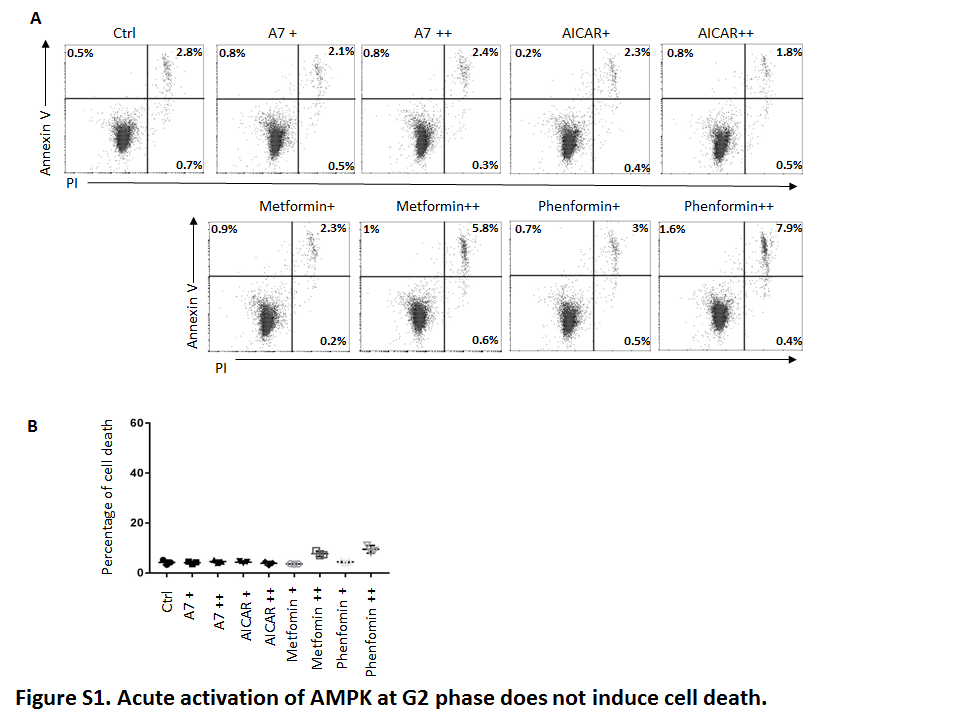

Supplement: Supporting Information [file supp_RA117.001379_134549_1_supp_66181_p3fj4f.tif]

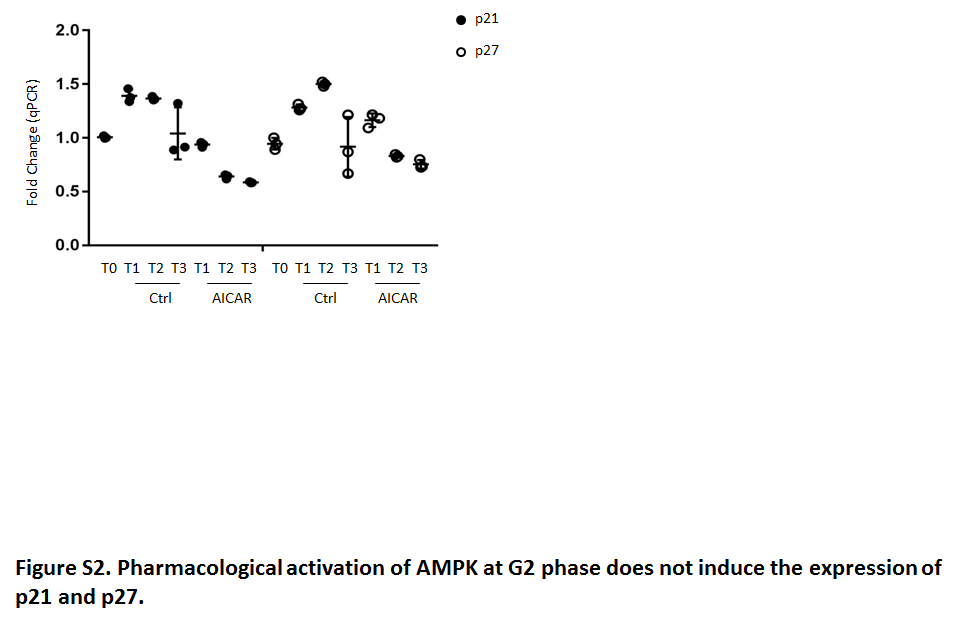

Supplement: Supporting Information [file supp_RA117.001379_134549_1_supp_66182_p3fj4f.tif]

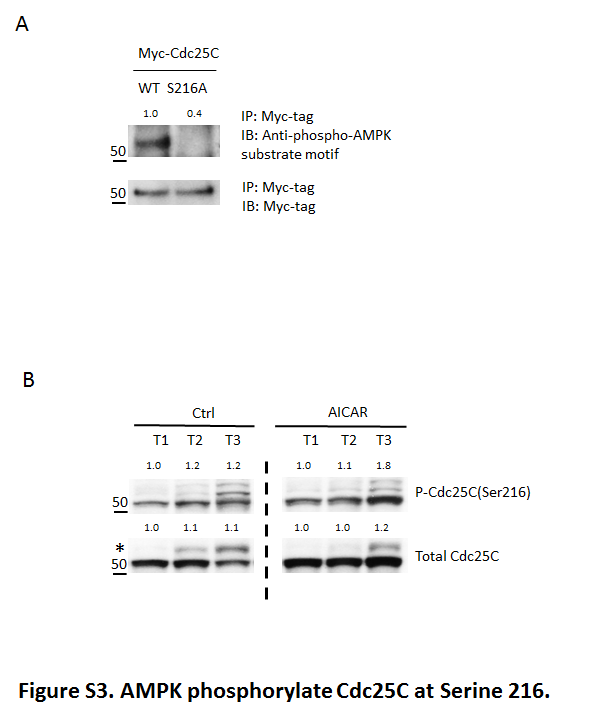

Supplement: Supporting Information [file supp_RA117.001379_134549_1_supp_66183_p3fj4f.tif]

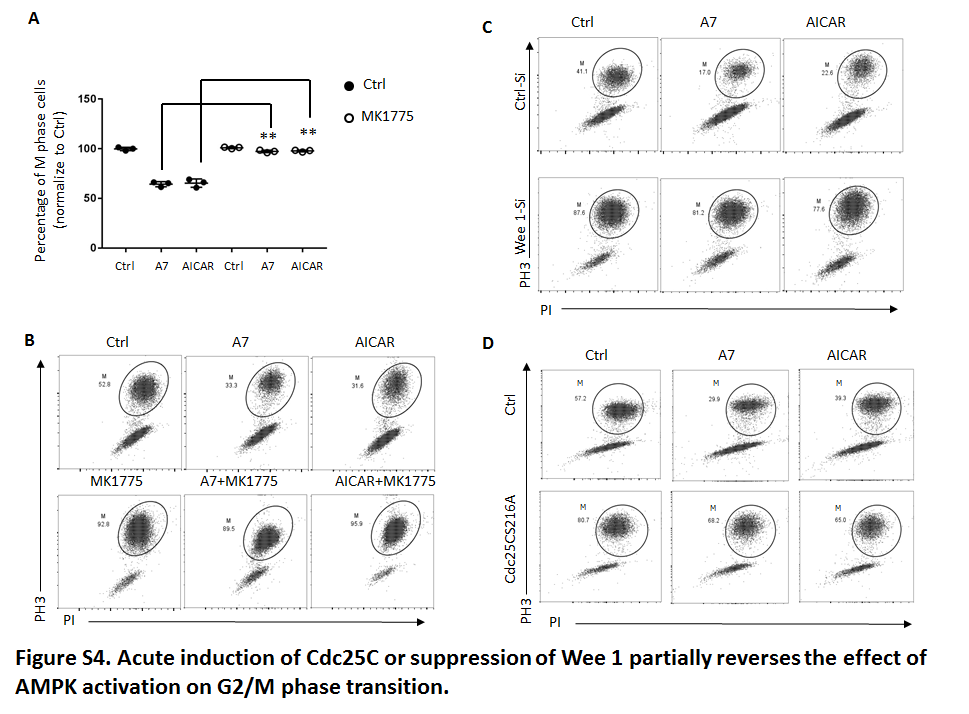

Supplement: Supporting Information [file supp_RA117.001379_134549_1_supp_66184_p3fj4f.tif]

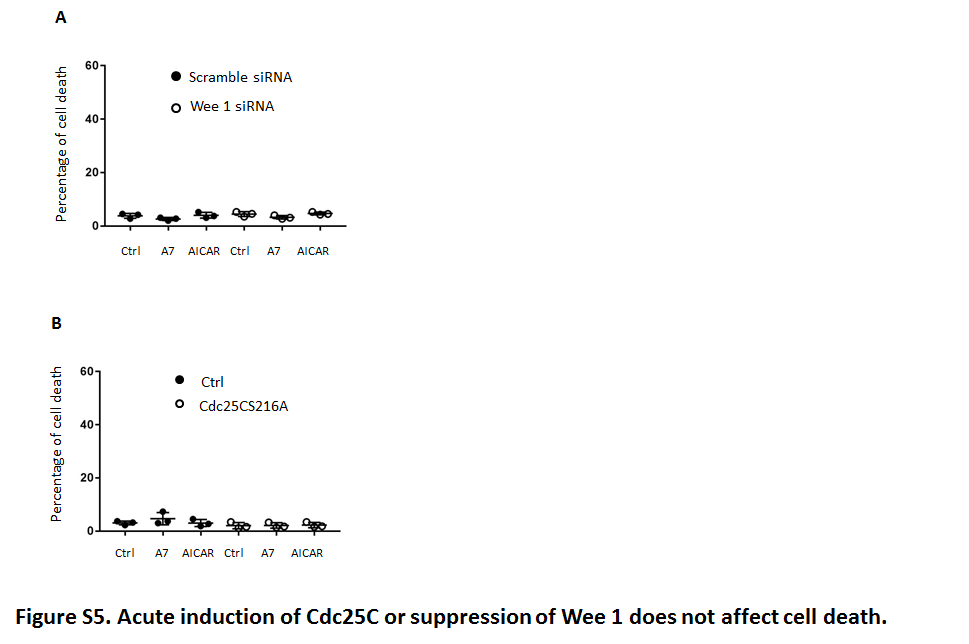

Supplement: Supporting Information [file supp_RA117.001379_134549_1_supp_66185_p3fj4f.tif]
