# Supplementary material for: Phosphorylation of CDC25C by AMP-activated protein kinase mediates a metabolic checkpoint during cell-cycle G2/M-phase transition
Source: J Biol Chem. 2018 Feb 21;293(14):5185–99. doi: 10.1074/jbc.RA117.001379 (PMC5892595; doi:10.1074/jbc.RA117.001379)
Supplement: Supporting Information [file supp_RA117.001379_134549_1_supp_66118_p3fj4f.docx]

**TABLE: S1**

| **Antibody identity** | **Catalog number** | **Source** |
| --- | --- | --- |
| phospho-AMPKα (Thr172) (40H9) Rabbit mAb | 2535 | Cell Signaling Technology |
| phospho-CDC25C (Ser216) antibody | 9528 | Cell Signaling Technology |
| phospho-CDC25C (Thr48) antibody | 9527 | Cell Signaling Technology |
| phospho-AMPK Substrate Motif [LXRXX(pS/pT)] MultiMab™ Rabbit mAb mix | 5759 | Cell Signaling Technology |
| CDC25C (5H9) mAb | 4688 | Cell Signaling Technology |
| Phospho-Chk1 (Ser345) (133D3) Rabbit mAb | 2348 | Cell Signaling Technology |
| Phospho-S6 Ribosomal Protein (Ser240/244) (D68F8) XP® Rabbit mAb | 5364 | Cell Signaling Technology |
| S6 Ribosomal Protein Rabbit mAb | 2217 | Cell Signaling Technology |
| Phospho-p70 S6 Kinase (Thr389) (108D2) Rabbit mAb | 9234 | Cell Signaling Technology |
| p70 S6 Kinase Rabbit mAb | 2708 | Cell Signaling Technology |
| Phospho-Histone H2A.X (Ser139) (20E3) Rabbit mAb | 9718 | Cell Signaling Technology |
| WEE1 (D10D2) Rabbit mAb | 13084 | Cell Signaling Technology |
| H2A.X Antibody (C-20) | sc-54606 | Santa Cruz Biotechnology |
| GST Antibody (56C1) | sc-80998 | Santa Cruz Biotechnology |
| Actin Antibody (2Q1055) | sc-58673 | Santa Cruz Biotechnology |
| c-myc antibody (9E10) | sc-40 | Santa Cruz Biotechnology |
| Chk1 Antibody (2G11D5) | sc-56288 | Santa Cruz Biotechnology |
| Anti-Thiophosphate ester antibody (51-8) | ab92570 | Abcam |

**TABLE: S2**

| **Genebank Accession#** | **Gene name** | **Primer-forward** | **Primer-reverse** |
| --- | --- | --- | --- |
| NM-004048 | β2microglobulin | GAGGCTATCCAGCGTACTCCA | CGGCAGGCATACTCATCTTTT |
| NM-078467 | Cdkn1a (p21) | TGTCCGTCAGAACCCATGC | AAAGTCGAAGTTCCATCGCTC |
| NM-004064 | CDKN1B (p27, Kip1) | AACGTGCGAGTGTCTAACGG | CCCTCTAGGGGTTTGTGATTCT |

**TABLE: S3**

| **Reagent** | **Catalog number** | **Source** |
| --- | --- | --- |
| [2-^14^C]-pyruvate | ARC0222 | American Radiolabeled Chemicals |
| [1-^14^C]-glucose | MC228 | Moravek |
| [5-^3^H] glucose | ART0115 | American Radiolabeled Chemicals |
| [9,10-^3^H]-palmitic acid | MT845 | Moravek |
| [U-^14^C]-glutamine | MC1124 | Moravek |
